# Equity in vaccine coverage in Uganda from 2000 to 2016: revealing the multifaceted nature of inequity

**DOI:** 10.1186/s12889-023-17592-6

**Published:** 2024-01-15

**Authors:** Anthony Ssebagereka, Gatien de Broucker, Elizabeth Ekirapa-Kiracho, Rornald Muhumuza Kananura, Alfred Driwale, Joshua Mak, Aloysius Mutebi, Bryan Nicholas Patenaude

**Affiliations:** 1 Makerere University School of Public Health, Kampala, Uganda; 2 Department of International Health, Johns Hopkins Bloomberg School of Public Health, Baltimore, USA; 3 International Vaccine Access Center, Johns Hopkins University, Baltimore, USA; 4 Uganda National Expanded Program On Immunization (UNEPI), Ministry of Health, Kampala, Uganda

**Keywords:** Vaccine equity, Vaccine coverage, Vaccine delivery, Access to healthcare, Uganda, Determinants of health, National medical stores, Immunization schedule, Zero-dose

## Abstract

**Background:**

This study analyses vaccine coverage and equity among children under five years of age in Uganda based on the 2016 Uganda Demographic and Health Survey (UDHS) dataset. Understanding equity in vaccine access and the determinants is crucial for the redress of emerging as well as persistent inequities.

**Methods:**

Applied to the UDHS for 2000, 2006, 2011, and 2016, the Vaccine Economics Research for Sustainability and Equity (VERSE) Equity Toolkit provides a multivariate assessment of immunization coverage and equity by (1) ranking the sample population with a composite direct unfairness index, (2) generating quantitative measure of efficiency (coverage) and equity, and (3) decomposing inequity into its contributing factors. The direct unfairness ranking variable is the predicted vaccination coverage from a logistic model based upon fair and unfair sources of variation in vaccination coverage. Our fair source of variation is defined as the child’s age – children too young to receive routine immunization are not expected to be vaccinated. Unfair sources of variation are the child’s region of residence, and whether they live in an urban or rural area, the mother’s education level, the household’s socioeconomic status, the child’s sex, and their insurance coverage status. For each unfair source of variation, we identify a “more privileged” situation.

**Results:**

The coverage and equity of the Diphtheria-Pertussis-Tetanus vaccine, 3^rd^ dose (DPT3) and the Measles-Containing Vaccine, 1^st^ dose (MCV1) – two vaccines indicative of the health system’s performance – improved significantly since 2000, from 49.7% to 76.8% and 67.8% to 82.7%, respectively, and there are fewer zero-dose children: from 8.4% to 2.2%. Improvements in retaining children in the program so that they complete the immunization schedule are more modest (from 38.1% to 40.8%). Progress in coverage was pro-poor, with concentration indices (wealth only) moving from 0.127 (DPT3) and 0.123 (MCV1) in 2000 to -0.042 and -0.029 in 2016. Gains in overall equity (composite) were more modest, albeit significant for most vaccines except for MCV1: concentration indices of 0.150 (DPT3) and 0.087 (MCV1) in 2000 and 0.054 and 0.055 in 2016. The influence of the region and settings (urban/rural) of residence significantly decreased since 2000.

**Conclusion:**

The past two decades have seen significant improvements in vaccine coverage and equity, thanks to the efforts to strengthen routine immunization and ongoing supplemental immunization activities such as the Family Health Days. While maintaining the regular provision of vaccines to all regions, efforts should be made to alleviate the impact of low maternal education and literacy on vaccination uptake.

**Supplementary Information:**

The online version contains supplementary material available at 10.1186/s12889-023-17592-6.

## Introduction

Vaccination is one of the most successful cost-effective interventions for controlling vaccine-preventable diseases, thereby reducing childhood morbidity and mortality [1]. Although remarkable improvement has been made since 1974 when the World Health organization introduced the expanded program for immunization, access to vaccines still remains a challenge in many developing countries with the lowest improvements reported among disadvantaged and marginalized groups. Furthermore, global coverage for vaccines has not changed markedly in the past decade. It dropped from 86% in 2019 to 83% in 2020. The drop in 2020 was largely attributed to disruptions caused by the Covid 19 pandemic. Uganda is one of the Gavi priority countries where the improvement of vaccination coverage and equitable uptake is a key concern. In the recent past, there has been a renewed call for governments and development partners to include strategic goals related to coverage and equity in their plans [2]. For instance, the Gavi and the Vaccine Alliance 5.0 (2021–2025) strategy has special focus on the unreached communities and under-immunized children with equity at the center of the strategy [3]. This is also reflected in the broad vision of the Immunization Agenda 2030 (IA2030) which states that: “A world where everyone, everywhere, at every age, fully benefits from vaccines for good health and well-being” [4].

The Uganda Expanded Programme on Immunization (UNEPI) has the mandate to ensure full immunization of children (aged 0–12 months) and women of childbearing age (15–45 years both pregnant and non-pregnant), and other high-risk groups as might be determined by the epidemiological patterns [5]. In Uganda, vaccination services are provided by MOH through UNEPI using the healthcare system structures. Furthermore, vaccination services in Uganda are managed at national, regional, district and community levels, with different stakeholders involved at each level. The Uganda National Expanded Programme on Immunization (UNEPI) and the Global Vaccine Action Plan set coverage goals of 90% at national level and 80% at district level by 2020. Overall, Uganda’s vaccination coverage estimates suggest good performance in 2019, especially for third dose Diphtheria, Tetanus and Pertussis (DPT3) at 93%, Pneumococcal Conjugate vaccine (PCV3) at 92%, Polio 3 at 92%, BCG at 88%, Rotavirus vaccines completed dose (RotaC) at 87%, and Measles-containing-vaccine first-dose (MCV1) at 87% [6].

However despite this overall relatively high coverage, inequities in access to vaccination has been noted and remains one of the biggest challenges in several districts of Uganda [7]. Such inequities should not be ignored, because persisting inequities in vaccine coverage can contribute to the frequent occurrence of outbreaks of vaccine preventable diseases [8]. Whereas studies done elsewhere have attributed similar inequities in vaccine coverage to factors such as household wealth, geographic location, maternal education, and child characteristics [9, 10], there is still a dearth of evidence in Uganda about the factors that contribute to inequities in vaccine coverage. Furthermore, there are also gaps related to the extent and types of inequity in vaccine coverage. Lack of evidence on the types of inequities and the explanatory factors that propagate such inequities constrains the development of appropriate and synchronized mitigation strategies and is therefore only likely to lead to persisting inequities in access to vaccines and poor child health outcomes that could have been prevented. This situation is further compounded by the fact that the administration of different vaccines and different doses of vaccines reflect different attributes of the immunization programme. For example, whereas DPT1 coverage is an indicator of health care access, DPT3 is an indicator of both health care access and the capacity to utilize immunization services on multiple visits.

Based upon this backdrop, this paper focuses on analysing vaccine coverage and equity among children under five years of age in Uganda based on data from the 2000, 2006, 2011, and 2016 Uganda Demographic and Health Surveys (UDHS). It will expand on the results from Okello et al. [11], which focused on the completion rate for children aged 12–23 months old. Strengthening equitable access to vaccines has potential to not only save lives, but also reduce morbidity, in addition to subsequently advancing economic development at both household and national levels. This will further facilitate the design and implementation of targeted programmes to overcome the identified indicators that explain variations in vaccine coverage and equity in Uganda.

## Methods

As detailed in our more extensive methodological paper [12] the VERSE composite vaccination equity assessment metric is derived from the work of Wagstaff and Erreygers on socioeconomic equity combined with measures of direct unfairness in healthcare access outlined in the works of Fleurbaey, Schokkaert, Cookson, and Barbosa [13–16].

The VERSE metric is a concentration index of vaccination coverage where individuals are ranked by multi-dimensional unfairness in access instead of using a measure of wealth alone. The direct unfairness ranking variable is the predicted vaccination coverage from a logistic (binary outcomes) model based upon fair and unfair sources of variation in vaccination coverage. Our fair source of variation is defined as the child’s age – children too young to receive routine immunization are not expected to be vaccinated. Unfair sources of variation are the child’s region of residence, and whether they live in an urban or rural area, the mother’s education level, the household’s socioeconomic status (estimated using a principal component analysis of the household’s reported assets [17]), the child’s sex, and their insurance coverage status. For each unfair source of variation, we identify a “more privileged” situation. This set of factors are used for the composite ranking and the decomposition analysis.

The produced equity metrics include the Wagstaff concentration index and its Erreyger correction [16, 18] (see the Supplementary Material), and the Absolute Equity Gap (AEG).

Concentration curves were calculated for all vaccine outcomes by mapping the cumulative proportion of vaccinated children against ranked population quintiles. The Wagstaff concentration indices correspond to twice the area between the concentration curve and the 45-degree line of perfect equality, which can be approximated by the curve’s covariance (see Eq. 1). Concentration indices ranging from 0 to 1 indicate a pro-privileged (composite ranking) or pro-rich (wealth-based ranking) distribution of vaccines, while concentration indices ranging from -1 to 0 indicate a pro-disadvantaged or pro-poor distribution of vaccines. While larger concentration indices indicate less equity in general, they are best interpreted in comparison: we compare concentration indices by year, vaccine, region/district, and ranking method (wealth only or composite). We computed concentration indices using socioeconomic status as the population ranking instead of the composite index to create equity measures comparable to other studies.

The AEG quantifies the (absolute) difference in vaccine coverage between the 20% most privileged and the 20% most disadvantaged people (see Eq. 3). Detailed coverage levels for the compared quintiles are presented in the Supplementary Material: Tables S18-21. A perfectly equitable distribution of vaccine – where unvaccinated children are randomly distributed across the different levels of the selected unfair factors – would have concentration indices and an AEG that equal zero.

1 $${CI}_{Wagstaff}=\frac{2}{{\mu }_{vc}}Cov({vci}_{direct},F({vci}_{du}))$$

Where ${\mu }_{vc}$ is the average vaccine coverage across the entire population and $Cov({vci}_{direct},F({vci}_{du}))$ the covariance between the directly standardized individual level of healthcare (the observed vaccination coverage) (${vci}_{direct}$) and the cumulative distribution function of direct unfairness ($F({vci}_{du})$). The Erreyger correction is calculated from the Wagstaff concentration index as follow (Eq. 2).

2 $${CI}_{Erreyger}=4\times {\mu }_{vc}\times {CI}_{Wagstaff}$$

3 $$AEG={vci}_{observed}\left(top\;20\%\;of\;F\left({vci}_{du}\right)\right)-{vci}_{observed}\left(bottom\;20\%\;of\;F\left({vci}_{du}\right)\right)$$

We can argue that the inequity is not statistically significant (with 95% confidence) when the 95% confidence interval overlaps zero (see Eqs. 4 and 5). When this is not the case, we assess that there is inequity driven by the selected unfair variables. Concentration indices are valuable when compared over time and across vaccine outcomes.

4 $$Upper\;and\;lower\;bound\;of\;the\;CI=CI\;\pm\;1.96\times\sqrt{Variance({vci}_{direct})}$$

5 $$Upper\;and\;lower\;bound\;of\;the\;AEG=AEG\pm1.96\times\sqrt{\left(\frac{s_{Q5}^2}{n_{Q5}}+\frac{s_{Q1}^2}{n_{Q1}}\right)}$$

Where ${n}_{Qx}$ is the number of observations in quintile $x$ and ${s}_{Qx}$ is the standard deviation.

A regression decomposition is performed to determine the relative share each unfair parameter has on overall inequality in vaccination status. Detailed methods can be found in our more extensive methodological paper [12].

The Uganda’s last four Demographic Health Survey (UDHS) is one of the major data sources for evidence regarding vaccine equity in Uganda. This analysis uses data from UDHS from 2000 to 2016 to assess equity for several statuses as part of Uganda’s immunization schedule (see Supplementary Material: Figure S1). These are immunization by Bacillus Calmette–Guérin vaccine (BCG), diphtheria-pertussis-tetanus vaccine doses 1 to 3 (DPT1-3), oral polio vaccine 1 to 3 (OPV1-3), measles containing vaccine first dose (MCV1), zero dose, fully immunized, and completely immunized. Zero dose status refers to a child who has received no vaccines on the national immunization schedule. Fully immunized means that a child has been fully immunized relative to their age. Complete immunization indicates that the child is older than 24 months of age and has completed their routine paediatric immunization schedule.

We focused the results below on the equity metrics generated for full immunization (*i.e.*, the child received all vaccines scheduled for their age), zero-dose status (*i.e.*, the child did not receive any vaccine), and DPT3 (*i.e.*, the child received their third dose of diphtheria-tetanus-pertussis). We recognize that coverage levels for DPT3 are generally widely accepted as standard indicators for performance of immunization system [19].

## Results

**Fig. 1:**
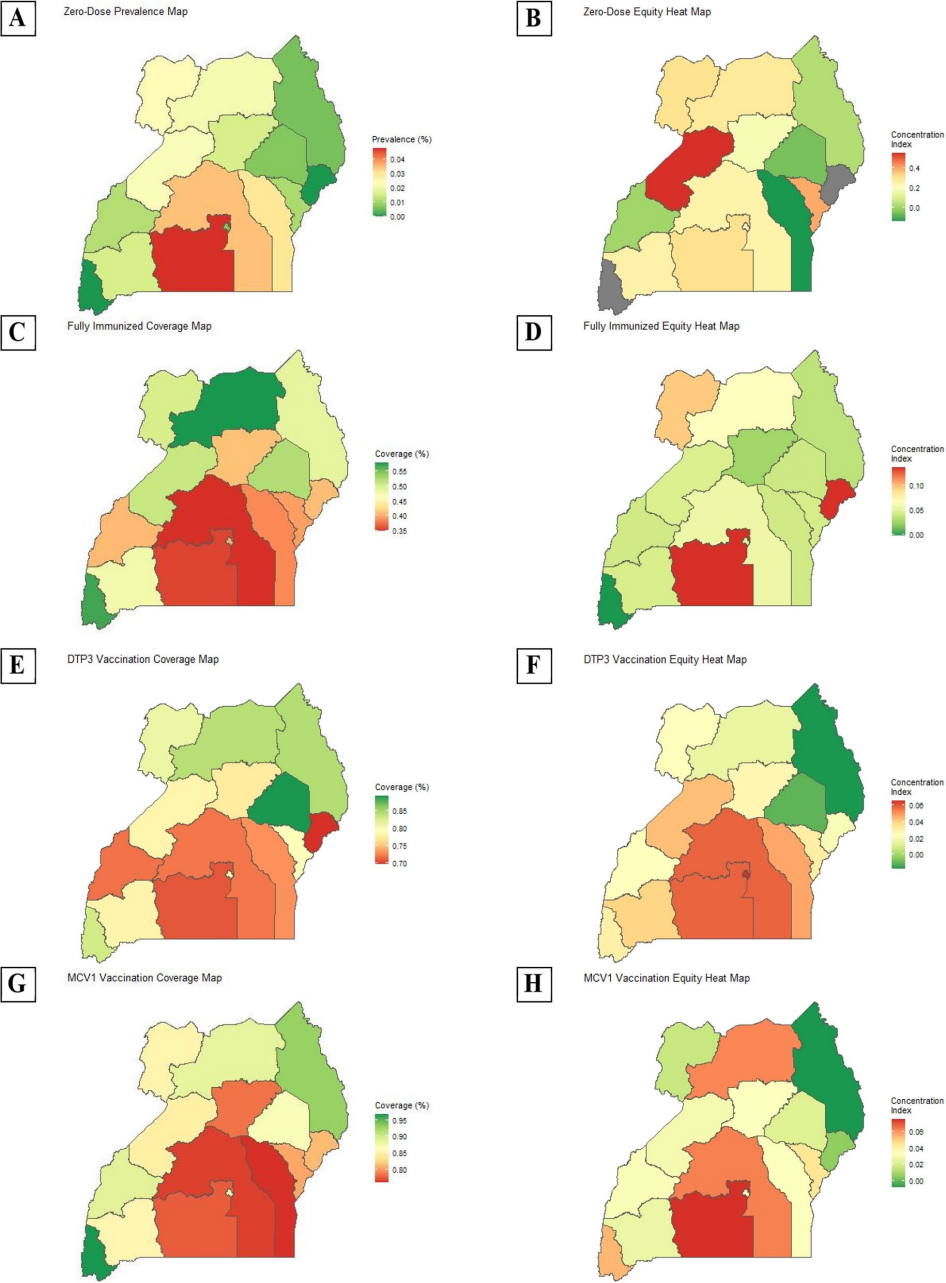
Vaccine coverage and equity maps for 2016. Map based on the 2016 DHS. The equity maps use the Wagstaff concentration index to present the level of inequity. Maps for the years 2000, 2006, and 2011 are presented in the Supplementary Material: Figures S2-S4. The panels are defined as such: **A** shows the map for the prevalence of zero-dose status among under-5 in 2016. **B** Shows the map for the level of inequity of zero-dose status among under-5 in 2016. **C** Shows the map for the coverage of full-immunization status among under-5 in 2016. **D** Shows the map for the level of inequity of full-immunization status among under-5 in 2016. **E** Shows the map for the coverage of the DPT3 vaccine (third dose) among under-5 in 2016. **F** Shows the map for the level of inequity of the DPT vaccine (third dose) among under-5 in 2016. **G** Shows the map for the coverage of the measles containing vaccine (first dose) among under-5 in 2016. **H** Shows the map for the level of inequity of the measles containing vaccine (first dose) among under-5 in 2016

**Fig. 2:**
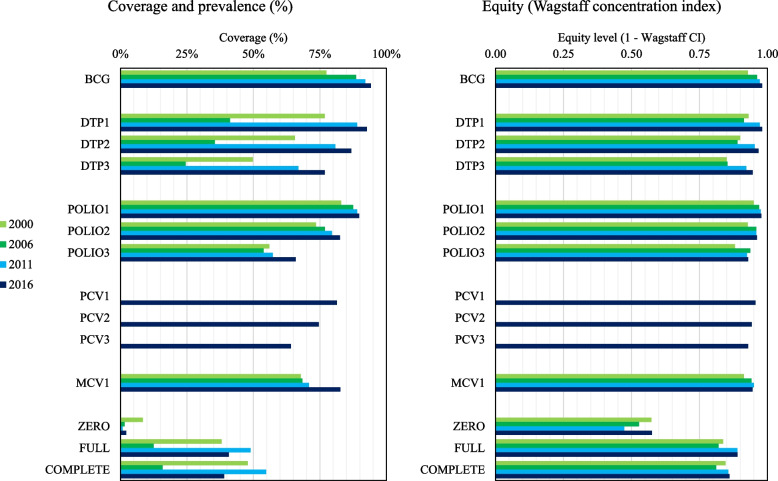
Evolution of vaccine coverage, health outcome prevalence, and equity from 2000 to 2016. Legend: PCV was introduced in 2013. Left graph presents the evolution of vaccine coverage and zero-dose status prevalence from 2000 to 2016 Right graph presents the evolution of the level of equity in vaccine coverage and zero-dose status prevalence from 2000 to 2016. The level of equity corresponds to 1 minus the absolute value of the Wagstaff concentration index

**Fig. 3:**
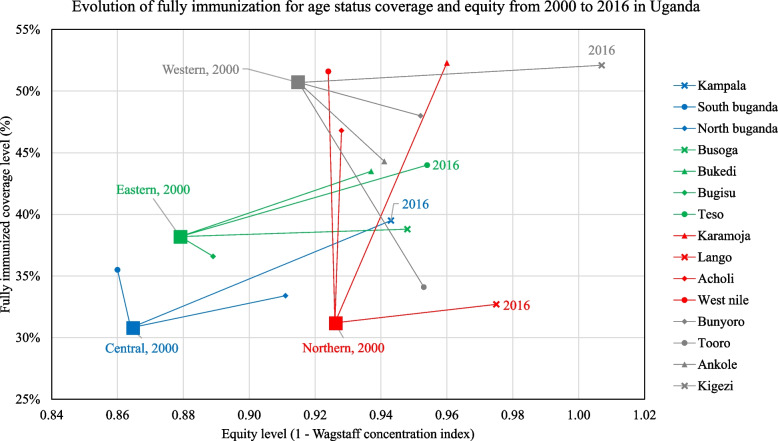
Equity-coverage plane for full immunization for age status by region from 2000 to 2016. Legend: Uganda changed the definition of its regions, disaggregating its four main regions (Central, Eastern, Northern, and Western) in 2000 into fifteen regions in 2016. The data points represent how the regions were defined in 2000 and 2016. We only labeled one endpoint for each series

**Fig. 4:**
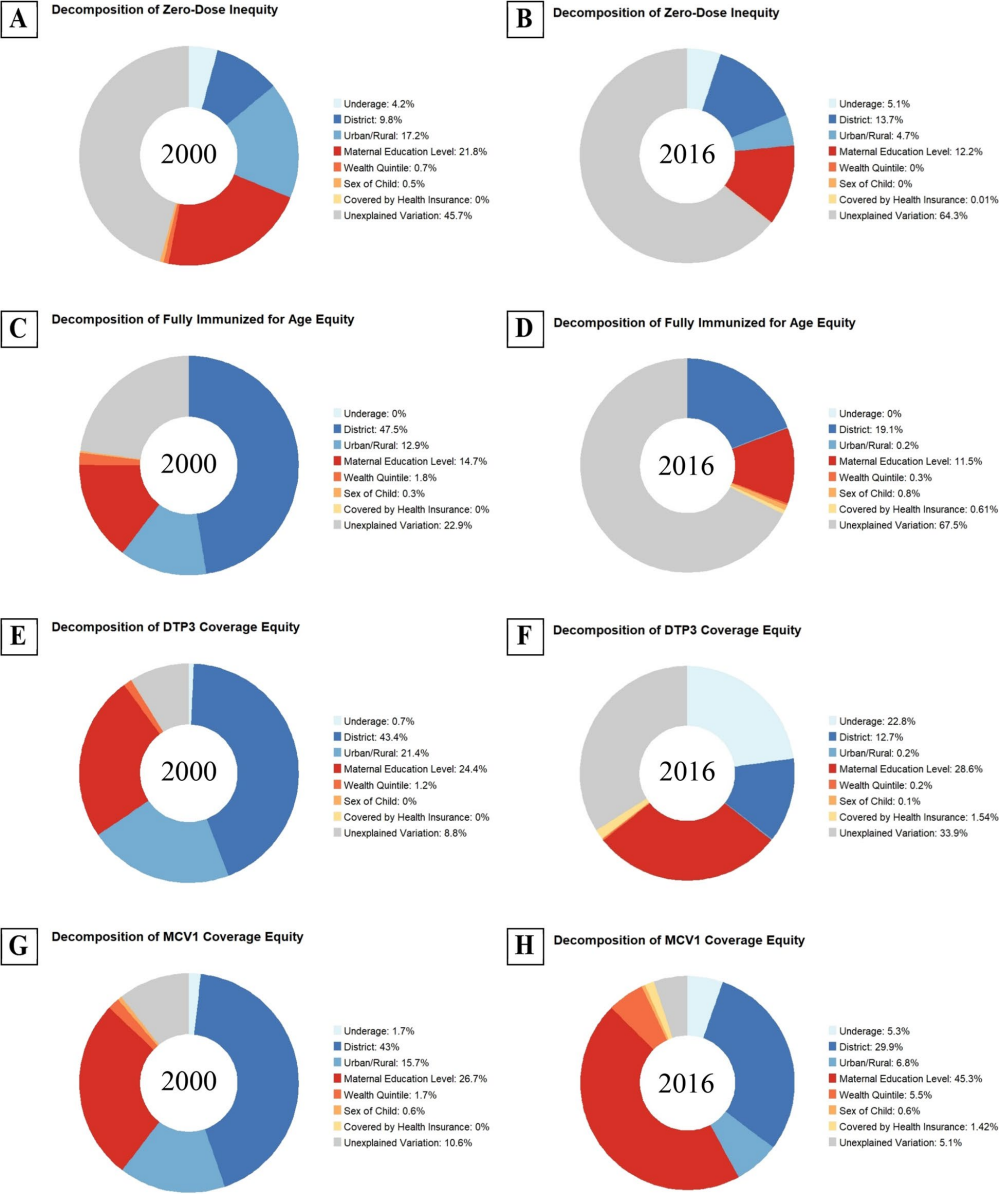
Effect of fair and unfair factors on vaccine coverage for fully immunized for age, DPT3 and MCV1, and zero-dose prevalence. Legend: Decomposition charts for the years 2000, 2006, and 2011 are presented in the Supplementary Material: Figures S5-S8. Children who were reportedly “underage” refers to children who are younger than 12 months and have not yet received any vaccine – they are considered at-risk of becoming “zero-dose” as per the Gavi definition. The panels are defined as such: **A** features the decomposition analysis for zero-dose status among under-5 in 2000. **B** Features the decomposition analysis for zero-dose status among under-5 in 2016. **C** features the decomposition analysis for full immunization (for age) in 2000. **D** Features the decomposition analysis for full immunization (for age) in 2016. **E** features the decomposition analysis for DPT vaccine (3^rd^ dose) in 2000. **F** Features the decomposition analysis for DPT vaccine (3^rd^ dose) in 2016. **G** Features the decomposition analysis for the measles vaccine (1^st^ dose) in 2000. **H** Features the decomposition analysis for the measles vaccine (1^st^ dose) in 2016

Table 1 summarizes the coverage and equity metrics (concentration index and absolute equity gap) for 2000 and 2016. Additional data for 2000, 2006, 2011, and 2016 is featured in Supplementary Material: Tables S1-16. Figure 1 shows the coverage and equity by region. Figure 2 tracks the evolution of national coverage and equity for all vaccines and health outcomes from 2000 to 2016. Figure 3 tracks the evolution of subnational coverage and equity for being fully immunized for age from 2000 to 2016. Figure 4 presents the decomposition analysis for zero-dose status and DPT3. Results for fully immunized for age and MCV1 are presented in the Additional file 1. Table S17 presents the level of coverage by sociodemographic characteristics.

**Table 1:** National-level coverage data

Vaccine or health outcome ^a^	Efficiency metric		Equity metrics					
	**Coverage or prevalence**		**Concentration index**				**Absolute Equity Gap (composite)**	
			Wealth only (Wagstaff)^b^		Composite (Wagstaff)			
	**2000**	**2016**	**2000**	**2016**	**2000**	**2016**	**2000**	**2016**
BCG	77.4% (76.4%, 78.4%)	94.2% (93.7%, 94,7%)	0.167 (0.162, 0.172)	-0.071 (-0.084, -0.058)	0.072 (0.064, 0.080)	0.020 (0.017, 0.023)	0.269 (0.240, 0.298)	0.098 (0.082, 0.114)
DPT1	76.8% (75.8%, 77.9%)	92.7% (92.1%, 93.2%)	0.157 (0.152, 0.162)	-0.062 (-0.075, -0.049)	0.070 (0.062, 0.078)	0.020 (0.016, 0.024)	0.234 (0.203, 0.265)	0.106 (0.084, 0.128)
DPT2	65.6% (64.4%, 66.8%)	86.8% (86.1%, 87.5%)	0.135 (0.129, 0.141)	-0.052 (-0.064, -0.040)	0.101 (0.090, 0.112)	0.033 (0.027, 0.039)	0.305 (0.270, 0.340)	0.146 (0.121, 0.717)
DPT3	49.7% (48.5%, 51.0%)	76.8% (75.9%, 77.8%)	0.127 (0.120, 0.134)	-0.042 (-0.053, -0.031)	0.150 (0.136, 0.164)	0.054 (0.046, 0.062)	0.358 (0.323, 0.393)	0.183 (0.154, 0.212)
POLIO1	83.0% (82.0%, 83.9%)	89.7% (89.1%, 90.4%)	0.173 (0.169, 0.177)	-0.058 (-0.070, -0.046)	0.051 (0.044, 0.058)	0.023 (0.018, 0.028)	0.185 (0.156, 0.214)	0.107 (0.083, 0.131)
POLIO2	73.5% (72.4%, 74.6%)	82.5% (81.7%, 83.4%)	0.144 (0.140, 0.148)	-0.051 (-0.063, -0.039)	0.073 (0.064, 0.082)	0.039 (0.032, 0.046)	0.261 (0.228, 0.352)	0.153 (0.126, 0.180)
POLIO3	56.0% (54.8%, 57.3%)	65.9% (64.9%, 67.0%)	0.117 (0.112, 0.122)	-0.048 (-0.059, -0.037)	0.120 (0.107, 0.133)	0.071 (0.061, 0.081)	0.317 (0.282, 0.352)	0.185 (0.154, 0.216)
PCV1	N/A	81.4% (80.6%, 82.2%)	N/A	-0.049 (-0.061, -0.037)	N/A	0.044 (0.038, 0.050)	N/A	0.177 (0.150, 0.204)
PCV2	N/A	74.6% (73.6%, 75.5%)	N/A	-0.043 (-0.054, -0.032)	N/A	0.058 (0.050, 0.066)	N/A	0.215 (0.186, 0.244)
PCV3	N/A	64.1% (63.1%, 65.2%)	N/A	-0.035 (-0.046, -0.024)	N/A	0.071 (0.061, 0.081)	N/A	0.197 (0.166, 0.228)
MCV1	67.8% (66.5%, 69.1%)	82.7% (81.7%, 83.7%)	0.123 (0.117, 0.129)	-0.029 (-0.040, -0.018)	0.087 (0.075, 0.099)	0.055 (0.045, 0.065)	0.234 (0.197, 0.271)	0.180 (0.149, 0.211)
ZERO^c^	8.4% (7.7%, 9.2%)	2.2% (1.8%, 2.5%)	0.236 (0.233, 0.239)	-0.078 (-0.091, -0.065)	0.427 (0.379, 0.475)	0.425 (0.341, 0.509)	0.123 (0.103, 0.143)	0.027 (0.019, 0.035)
FULL	38.1% (36.8%, 39.3%)	40.8% (39.7%, 41.8%)	0.133 (0.127, 0.139)	-0.043 (-0.054, -0.032)	0.163 (0.145, 0.181)	0.110 (0.096, 0.124)	0.291 (0.254, 0.328)	0.230 (0.199, 0.261)
COMPLETE	47.9% (46.1%, 49.6%)	39.0% (37.3%, 40.8%)	0.133 (0.124, 0.142)	-0.044 (-0.063, -0.025)	0.154 (0.134, 0.174)	0.140 (0.114, 0.166)	0.363 (0.312, 0.414)	0.267 (0.214, 0.320)

^a^ZERO, the child didn’t receive any vaccine by 12 months old; FULL, the child is under 24 months old and is fully immunized for their age; COMPLETE, the child is above 24 months and completed the routine paediatric immunization schedule

^b^Concentration index based on households ranked by socioeconomic status (as defined in the DHS) only

^c^For mathematical reasons, when the prevalence/coverage outcome is low (in this case, 2.2%), the Wagstaff and Erreyger indices may produce conflicting results in terms of order of magnitude: the Wagstaff index reported a value of 0.425 (significant inequity) whereas the Erreyger corrected index was 0.022 (very equitable distribution). Both indices are positive: privileged people benefit most

### Zero-dose status

Zero-dose refers to a child not receiving a single dose of national immunization schedule vaccine by 12 months of age. A very small proportion (2.2%) of all children in Uganda have never received any vaccine according to the UDHS 2016, as compared to 8% captured in the UDHS 2000. The AEG showed an achievement gap of 0.027%, which shows a modest inequity between the 20% most disadvantaged and 20% most privileged households in Uganda. The extreme value for the Wagstaff concentration index (0.425; significant inequity) indicates that nearly all children with zero-dose status are in the most disadvantaged group.

On the other hand, it was observed that concentration indices for zero-dose status between UDHS 2000 and 2016 was comparable at 0.57 and 0.58 respectively. An all-time low concentration index for zero-dose status was registered in the UDHS 2011 at 0.47.

Decomposition of the inequity for UDHS 2016 found that the region (13.7%) where the household resides affected most whether a child had ever received any vaccine, closely followed, by maternal education (12.2%), and whether the household resided in an urban or rural community (4.7%). Being underaged for vaccination (a *fair* contributor) also contributed to 5.1% of the variation and here refers to children who are younger than 12 months old and have not received any vaccines yet: they are at risk of becoming “zero-dose” as per Gavi’s definition. On the other hand, the decomposition of the inequity for UDHS 2000 found that maternal education (21.8%), the setting where the household is located in an urban or rural community (17.2%), and the region (9.8%) were the major contributors of a child ever receiving any vaccine. Neither the sex of the child, nor the household’s socioeconomic status or health insurance coverage had any impact on whether the child had a zero-dose status. About 64% of the variation was left unexplained in UDHS 2016 compared to 45.7% for UDHS 2000.

South (5%) and North Buganda (4%) reported the highest prevalence of zero-dose children, with an unequal distribution, with a Wagstaff concentration index of 0.312 and 0.254 respectively. Most other regions had a zero-dose prevalence ranging between 0 and 3%, but with a large range of concentration indices: 0 to 0.553. The lowest level of equity was found in Bunyoro (0.553), and this was also the only region with a higher index than South Buganda. Two regions had negative concentration indices, indicating that less children among the most disadvantaged households were zero-dose compared to more privileged households: Busoga, with a zero-dose prevalence of 3% and a concentration index of -0.142, and Teso, with a prevalence of 1% and an index of -0.055.

### DPT3 coverage

Generally, National DPT3 coverage improved from 50% (UDHS 2000) to 76.8% (UDHS 2016). However, the lowest DPT3 coverage was experienced in UDHS 2006 at 25%. Six of the fifteen UDHS regions in UDHS 2016 had a DPT3 coverage level at or above 80% (none above 90%), with Teso and Karamoja displaying the highest DPT3 coverage levels (85% and 89%, respectively). South and North Buganda, Tooro, and Busoga had the lowest DPT3 coverage, estimated between 71 and 73%.

There was an improvement in concentration indices from 0.85 (UDHS 2000) to 0.95 (UDHS 2000). Due to its lower coverage, DPT3 vaccination distribution is less equitable than for DPT1, BCG, and OPV1. The Wagstaff concentration index was 0.054. The AEG was 0.183: the 20% most disadvantaged people would need to increase DPT3 vaccine coverage by 18.3% to have similar levels to the top 20% most privileged. Based on the Wagstaff index, Kampala (0.066), followed by South and North Buganda (both 0.059) had lower levels of distributional equity than the other regions.

Considering UDHS 2016, several unfair factors of variation also had a dominant influence: the level of maternal education (28.6%), followed by being underaged to receive the DPT3 vaccine (22.8%), and the region of residence (12.7%). On the other hand, in the UDHS 2000, region (43.4%), level of maternal education (24.4%) and urban/rural setting where household is located (21.4%) were the major unfair factors of variation in DPT3 coverage.

When computing the (more traditional) concentration index based on wealth only, it returned positive, or “pro-rich”, values for all vaccines and statuses in the earliest UDHS in 2000, and negative, or “pro-poor”, values afterward. This indicator reveals that the distribution of vaccines and their associated outcomes in Uganda benefited most wealthier people in 2000, and that this trend was reversed in the following years: now poorer Ugandans benefit most (although only slightly more) from it. While it displays an encouraging trend, using such wealth-based indicators may skew our assessment of distributional equity: adding the other demographical, social, and economic factors through the VERSE Equity Toolkit (detailed in the Methods) brings the concentration indices back in positive values, highlighting the adverse effect of compounding disadvantages on coverage.

## Discussion

We conducted an analysis to estimate variations in vaccine coverage and equity in Uganda, based on data collected from the UDHS of 2000, 2006, 2011 and 2016. We found that hardly a third of all the children under 24 months old in Uganda were fully immunized for age, and that there was inequity in full vaccination status, with the under-privileged populations being most affected as portrayed by the high AEG estimate. On the other hand, we found that a very small proportion of all children in Uganda had not received any of the nationally scheduled immunization vaccines by 12 months of age. It was also observed that the influence of the household’s residence (the district and whether urban or rural) on the inequitable distribution of vaccines significantly reduced between 2000 to 2016.

The households’ behaviour, whether guided by financial and social constraints, awareness, or beliefs, seemed to play a stronger role in immunization, particularly in 2016. Our findings are consistent with the findings from two other studies conducted in Uganda and Ethiopia that reported that maternal education, wealth status, region of residence, and the household head’s sex were strongly associated with full vaccination coverage [9, 11]. Strengthening the capacity of the health systems to vaccinate priority populations is critical to improving coverage and equity. The kind of evidence generated by our study and other similar studies on vaccine equity is therefore critical for improving the coverage and impact of immunization programs [20].

### Inequity in full immunization for age versus complete immunization—Children dropping out of the EPI

Childhood vaccination is an essential public health intervention that can significantly lower disparity levels towards a perfect equity stance [21]. Completion of the immunization schedule is crucial to achieving low childhood morbidity and mortality. Although the Ugandan Government has taken steps to increase access to vaccination services by providing them free to the clients, as noted above inequities in full and complete immunization persist. Generally, there was an improvement in the proportion of children who were fully vaccinated for age (“FULL” in Table 1) and those 24 months old and older that had completed their vaccination schedules (“COMPLETE”) comparing UDHS 2000 and UDHS 2016. Our analysis noted that there was a drop in the proportion of children that were fully immunized for age (45.7%) and the proportion that completed the routine paediatric immunization schedule by the age of 24 months (43.4%) implying that there are additional barriers that hinder completion of immunization. Furthermore, the Wagstaff and Erreyger corrected concentration indices both indicated inequity in full vaccination status with the status more prevalent amongst more privileged people. The sharp drop in the proportion of children that were fully vaccinated for age during UDHS 2006 could have been influenced by a number of factors including aspects of cost of implementation of the vaccination programs, increment in the facility catchment populations and shortages in human resources for health [22]. This drop cannot be well explained by the typical contributors to inequity included in the VERSE toolkit (see Supplementary Material: Figure S6). Similarly to children who do not receive any vaccine (zero-dose), the low proportion of children fully immunized for their age could be partly explained by variations relating to access to vaccination services and missed opportunities for vaccination [9].

There was a reduction in the equity gap between the UDHS 2000 and UDHS 2016. Uganda is one of the countries globally implementing the highest number of pro-equity immunization strategies (over 38 strategies on both the demand- and supply-side) [23], which have subsequently been a large contribution in bridging the equity gaps in immunization programming between UDHS 2000 and 2016. Hence, Uganda is currently ranked as a Gavi Tier-1 country with initial self-financing [24].

Looking at the coverage for BCG, DPT1 and OPV1 which are given at birth and DPT3 and OPV3 which are given at 14 weeks, we see a clear drop for the later doses, particularly between the 2nd and 3rd dose for both vaccines. This highlights the fact that whereas access for the first dose which is given at birth is relatively good, several challenges hinder continued access to the vaccines leading to the observed dropouts seen. As reported by Sodha et al., this is likely to be the result of supply side challenges related to poor infrastructure for vaccines regarding the supply chain and human resource as well as demand side factors that lead to vaccine hesitancy stemming from factors such as fears about vaccine safety and side effects as well as administration of multiple vaccines [19]. The measles vaccine which is received at 9 months however has a higher coverage than OPV3 and DPT3. The higher measles vaccination may be attributed to mass vaccination campaigns that are not usually done for the other two vaccines [25].

The variables that strongly contribute to inequity for fully immunized children for age included the district of residence and maternal education. Other studies report that employment, ante-natal care follow-up, wealth quintile, and delivery at the health facility also do contribute to full immunization [26]. Instead of these specific parameters for predicting vaccination inequity among the children who were fully immunized for age, our study used maternal education as it is documented to impact both the mother and the child’s health: lower maternal education is associated with decreased used of preventive care and childcare, slower development of motor and cognitive skills, and generally higher mortality risk [27–29]. The significant unexplained variation (66%) of inequity for fully immunized children for age implicates the existence of other factors unrelated to maternal education and the other selected parameters. A factor that was raised by district health officers (private communications) surround increases in vaccine hesitancy among Ugandans of all sociodemographic status and education level. While we cannot easily draw conclusions on whether the household was influenced by anti-vaccine beliefs through the DHS data, we should consider it as a likely significant contributor to demand-side constraints.

If vaccine hesitancy were associated with maternal education, the most salient example of it from the DHS data would be the strong association between the measles vaccine (MCV1) coverage and maternal education, particularly in 2016. The region of residence forms the most (in 2000) and then second most (2016) dominant factor, which can indicate either shortcomings in vaccine supply or a population cluster effect on coverage. Efforts to improve MCV1 coverage in specific districts or target population would reduce the occurrence of outbreaks that have been noted despite high vaccination coverages and attributed to inequity and drops in coverage among other factors [30].

### High and low performing regions

The north and south Buganda had the lowest rates of achievement for full vaccination. The central region (north and south Buganda), includes Kampala, the capital city of Uganda which grapples with unique challenges including having a large population of urban informal settlement dwellers (especially the urban-poor) as well as new populations with widely varying health seeking behaviour and limited access to public health services [7]. For example, it is sometimes argued that given the nature of their work, several caretakers fail to apportion time to take their children for immunization [7] leading to inequitable access to immunization services [31]. In addition to strengthening routine immunization, the government in partnership with UNICEF and other NGOs implemented supplementary immunization activities, such as Family Health Days, which significantly improved the coverage of DPT3 and MCV1 in the last decade [7].

On the other hand, Bugisu and south Buganda regions were the least equitable. Some of the reasons that could have contributed to this finding include the fact that South Buganda has several Island districts which are hard-to-reach areas, including Kalangala and Buvuma districts. The populations in these districts are largely underserved, due to limited resources (such as boats, fuel, health workers, outreach staff to mobilize and do health education, etc.) coupled with the long travel time required to traverse the islands and unpredictable weather patterns on the lake. These bottlenecks consequently pose enormous challenges for equitable access to vaccination services for the island communities compared to their counterparts on the mainland. On the other hand, Bugisu region also has hard-to-reach populations in the mountainous terrains in some districts located in the Elgon Mountain ranges, such as Manafwa, Bududa and Sironko, among others. These districts are also faced with unique supply-related bottlenecks that include inadequate health facilities and health workers in the hard-to-reach areas as well as poor access roads for ease of navigation within and between communities in addition to limited access to appropriate safe transport especially in the mountainous areas. Mass campaigns may be better capable of serving under-privileged populations in such hard to reach areas compared to the routine immunization system which is challenged by several bottlenecks [25].

We also noted that the Acholi region had fairly good coverage for MCV1, but rather low concentration indices, which implies that the under-privileged populations in that region have low access to vaccination services. Some of the factors that could have contributed to this variation include aspects of maternal education, district and residence type (urban/rural) in addition to supply side challenges such as long distance and poor access to healthcare services.

On the other hand, the Karamoja region which is a typically hard to reach region with nomadic pastoralists had consistently good coverage and equity for the MCV1, which is an indication that the interventions that were introduced to address supply and demand side bottlenecks have been successfully implemented. Facing a resurgence of vaccine-preventable human (*e.g.,* yellow fever) and animal diseases (*e.g.,* Peste des petits ruminants) due to increased sedentarism, efforts were recently made to deliver both human and animal vaccines to Karamoja [32, 33]. According to Mupere et al. [7], strengthened routine immunization in Karamoja allowed the region to achieve good coverage before Family Health Days were implemented in 2011 – a notable difference from other Ugandan regions.

### Maternal education and vaccine coverage

Maternal level of education is a powerful predictor of higher full vaccination and completion of the immunization schedule [31, 34]. Our analysis suggests that maternal education, to a larger extent, explains variations of inequity in vaccine coverage, based on the UDHS data. These findings concur with those reported in a systematic review done by Rainey et al., who also reported that low education levels for the maternal caregiver was one of the most commonly identified reasons for under-vaccination [35]. Vaccination coverage has been observed by some scholars to have a regressive distribution with respect to maternal education, with relatively higher vaccination coverage among children whose mothers had higher maternal education compared to those whose mothers had lower maternal education [9, 36]. This therefore suggests that vaccination programs need to give critical attention to those with low formal education [34].

Our findings were also in conformity with a systematic review done in six countries that reported that maternal education inequalities were, in several instances, more profound than household wealth-based inequalities [37]. For instance, the systematic review reported that variations in maternal education created much larger inequalities in vaccine coverage as compared to household wealth differences in the countries of Nepal, Afghanistan and Pakistan. It is also important to note that higher levels of maternal education provide opportunity to raise awareness about the importance of vaccination thereby boosting uptake and vaccination coverage [9]. Low maternal education levels could be responsible for the poor attitude and low uptake of immunization services, especially in the regions faced with the large inequities [7].

Other similar studies report that disparities in full immunization coverage varied considerably by maternal education status, from 31% among those with no education at all, to 72% among those with secondary education or a higher level of education [20].

Policymakers and implementing partners need to prioritize policies and interventions to reduce inequities in vaccination coverage, with special focus on improving maternal education, and narrow equity gaps relating to variations in districts, residence type as well as region (between and within) in an effort to improve full vaccination coverage.

### Limitations of the analysis

We were not able to triangulate or deepen our findings with additional interviews which would have provided more insights into some of the unexplained variations. For instance, qualitative interviews would have provided some additional information on the system level indicators/bottlenecks that could have influenced the inequities in vaccine coverage that were observed. Furthermore, the UDHS data collected information mainly on caregiver, child, and household related indicators and much less information about the healthcare system level indicators. We attempted to relate/triangulate our findings with literature from similar studies conducted in other settings, in an attempt to bridge this gap.

## Conclusion

Significant improvements in both vaccine coverage and equity occurred in the past two decades, likely thanks to the government and its partners’ efforts to strengthen routine immunization, and the various supplementary immunization activities. The latter, through vaccination campaigns and the Family Health Days started in 2011, improved coverage for DTP3 and MCV1 in several districts. In others, like Karamoja, an opportunity to strengthen routine immunization came sooner with an increased sedentarism, low vaccine coverage and the need to respond to ensuing outbreaks.

Those efforts tackled supply constraints and significantly reduced differences across districts in vaccine coverage or, at least, their contribution to the remaining inequity in coverage. Most vaccines, with the notable exception of MCV1, saw a shift in the determinants of inequity where the factors generally associated with supply-side constraints – district and settings of residence – decreased, while demand-side constraints increased or remained unchanged, thus becoming the main contributor to vaccine coverage inequity. Maternal education by far has a dominant influence on equity, suggesting that developing appropriate public health communication and facilitating access to healthcare for populations with lower literacy levels may have the greatest effect.

The specific opportunities identified in this equity analysis for reducing inequity included the need to target the districts/regions with highest inequity, a focus on increasing maternal education as a fulcrum for boosting vaccine coverage and equity, as well as prioritizing delivery of vaccines to the urban-poor populations. Implementers should also consider expanding the scope of variables collected during immunization coverage surveys to broaden the range of independent variables that can be included during analysis to explain observed variations in inequity to vaccines.

### Supplementary Information

**Additional file 1: Figure S1.** Uganda EPI schedule (2019). **Table S1.** National-level coverage data for 2016. **Table S2.** National-level coverage data for 2011. **Table S3.** National-level coverage data for 2006. **Table S4.** National-level coverage data for 2000. **Figure S2.** Vaccine coverage and equity maps for 2011. **Figure S3.** Vaccine coverage and equity maps for 2006. **Figure S4.** Vaccine coverage and equity maps for 2000. **Figure S5.** Effect of fair and unfair factors on zero-dose prevalence from 2000 to 2016. **Figure S6.** Effect of fair and unfair factors on being fully immunized for age from 2000 to 2016. **Figure S7.** Effect of fair and unfair factors of DPT3 vaccine coverage from 2000 to 2016. **Figure S8.** Effect of fair and unfair factors on zero-dose prevalence from 2000 to 2016. **Table S5.** Subnational vaccine coverage rates for 2016. **Table S6.** Subnational vaccine coverage rates for 2011. **Table S7.** Subnational vaccine coverage rates for 2006. **Table S8.** Subnational vaccine coverage rates for 2000. **Table S9.** Subnational vaccine equity metric for 2016: Wagstaff multivariate concentration index. **Table S10.** Subnational vaccine equity metric for 2011: Wagstaff multivariate concentration index. **Table S11.** Subnational vaccine equity metric for 2006: Wagstaff multivariate concentration index. **Table S12.** Subnational vaccine equity metric for 2000: Wagstaff multivariate concentration index. **Table S13.** Subnational vaccine equity metric for 2016: Erreyger multivariate concentration index. **Table S14.** Subnational vaccine equity metric for 2011: Erreyger multivariate concentration index. **Table S15.** Subnational vaccine equity metric for 2006: Erreyger multivariate concentration index. **Table S16.** Subnational vaccine equity metric for 2000: Erreyger multivariate concentration index. **Table S17.** National coverage estimates by sociodemographic group in 2016. **Table S18.** National coverage estimates by sociodemographic group in 2000. **Table S19.** Coverage estimates by composite quintile and absolute equity gap in 2016. **Table S20.** Coverage estimates by composite quintile and absolute equity gap in 2011. **Table S21.** Coverage estimates by composite quintile and absolute equity gap in 2006. **Table S22.** Coverage estimates by composite quintile and absolute equity gap in 2000. 

## Data Availability

The datasets supporting the conclusions of this article are available in the Demographic and Health Surveys repository: ▪ Uganda: Standard DHS, 2016: https://dhsprogram.com/methodology/survey/survey-display-504.cfm (last accessed on 18 July 2022). ▪ Uganda: Standard DHS, 2011: https://dhsprogram.com/methodology/survey/survey-display-399.cfm (last accessed on 18 July 2022). ▪ Uganda: Standard DHS, 2006: https://dhsprogram.com/methodology/survey/survey-display-266.cfm (last accessed on 18 July 2022). ▪ Uganda: Standard DHS, 2001: https://dhsprogram.com/methodology/survey/survey-display-169.cfm (last accessed on 18 July 2022). The VERSE Equity Toolkit used for the analysis is available in open access: Patenaude, Bryan, de Broucker, Gatien, Mak, Joshua, Sriudomporn, Salin, Odihi, Deborah. 2022, "Official VERSE Equity Toolkit GitHub Repository to analyze DHS data", https://github.com/VERSE-Equity/Toolkit-DHS, GitHub, last accessed on 26 October 2023.

## Abbreviations

BCG: Bacille Calmette-Guérin Vaccine
DPT: Diphtheria-Pertussis-Tetanus Vaccine
DPT1: First dose of Diphtheria-Pertussis-Tetanus Vaccine
DPT2: Second dose of Diphtheria-Pertussis-Tetanus Vaccine
DPT3: Third dose of Diphtheria-Pertussis-Tetanus Vaccine
EPI: Expanded Program for Immunization
IPV: Inactivated Polio Vaccine
MCV: Measles-Containing Vaccine
MCV1: First dose of Measles-Containing Vaccine
NGO: Non-Governmental Organization
OPV: Oral Poliovirus Vaccine
PCV: Pneumococcal Conjugate Vaccine
PCV1: First dose of Pneumococcal Conjugate Vaccine
PCV2: Second dose of Pneumococcal Conjugate Vaccine
PCV3: Third dose of Pneumococcal Conjugate Vaccine
POLIO: Poliovirus Vaccine (either IPV or OPV)
POLIO1: First dose of Poliovirus Vaccine (either IPV or OPV)
POLIO2: Second dose of Poliovirus Vaccine (either IPV or OPV)
POLIO3: Third dose of Poliovirus Vaccine (either IPV or OPV)
UDHS: Uganda Demographic and Health Survey
UNEPI: Uganda National Expanded Program for Immunization
VERSE: Vaccine Economics Research for Sustainability and Equity project
