# Supplementary material for: Equity in vaccine coverage in Uganda from 2000 to 2016: revealing the multifaceted nature of inequity
Source: BMC Public Health. 2024 Jan 15;24:185. doi: 10.1186/s12889-023-17592-6 (PMC10790460; doi:10.1186/s12889-023-17592-6)
Supplement: Supplementary file 1 — Additional file 1: Figure S1. Uganda EPI schedule (2019). Table S1. National-level coverage data for 2016. Table S2. National-level coverage data for 2011. Table S3. National-level coverage data for 2006. Table S4. National-level coverage data for 2000. Figure S2. Vaccine coverage and equity maps for 2011. Figure S3. Vaccine coverage and equity maps for 2006. Figure S4. Vaccine coverage and equity maps for 2000. Figure S5. Effect of fair and unfair factors on zero-dose prevalence from 2000 to 2016. Figure S6. Effect of fair and unfair factors on being fully immunized for age from 2000 to 2016. Figure S7. Effect of fair and unfair factors of DPT3 vaccine coverage from 2000 to 2016. Figure S8. Effect of fair and unfair factors on zero-dose prevalence from 2000 to 2016. Table S5. Subnational vaccine coverage rates for 2016. Table S6. Subnational vaccine coverage rates for 2011. Table S7. Subnational vaccine coverage rates for 2006. Table S8. Subnational vaccine coverage rates for 2000. Table S9. Subnational vaccine equity metric for 2016: Wagstaff multivariate concentration index. Table S10. Subnational vaccine equity metric for 2011: Wagstaff multivariate concentration index. Table S11. Subnational vaccine equity metric for 2006: Wagstaff multivariate concentration index. Table S12. Subnational vaccine equity metric for 2000: Wagstaff multivariate concentration index. Table S13. Subnational vaccine equity metric for 2016: Erreyger multivariate concentration index. Table S14. Subnational vaccine equity metric for 2011: Erreyger multivariate concentration index. Table S15. Subnational vaccine equity metric for 2006: Erreyger multivariate concentration index. Table S16. Subnational vaccine equity metric for 2000: Erreyger multivariate concentration index. Table S17. National coverage estimates by sociodemographic group in 2016. Table S18. National coverage estimates by sociodemographic group in 2000. Table S19. Coverage estimates by composite quintile and absolute equity [file 12889_2023_17592_MOESM1_ESM.docx]

**Supplementary material for Equity in vaccine coverage in Uganda**

**Additional results**

*Full immunization for age*

A child was considered fully immunized if they received all scheduled vaccines for their age. Overall, there was an improvement in the proportion of children who were fully vaccinated for age from 38% (UDHS 2000) to 41% (UDHS 2016). However, there was a sharp drop in the percentage of children that were fully vaccinated for age in UDHS 2006, falling to just 13%. We also noted that there was a decline in the proportion of children that were completely vaccinated from 48% (UDHS 2000) to 39% (UDHS 2016).

The Wagstaff concentration index was 0.111, indicating inequity in full vaccination status, as this status is more prevalent amongst more privileged people. The AEG of 0.188 implied that the 20% most disadvantaged households would need to increase full immunization status by 18.8% to have similar levels to the top 20% most privileged households. Similarly, an AEG of 0.233 for immunization completion implied that the 20% most disadvantaged households would need to increase immunization completion status by 23.3% to have similar levels to the top 20% most privileged households. Overall, there was an improvement in concentration index concentration indices for full vaccination between UDHS 2000 and that of 2016 rising from 0.84 to 0.89 respectively. In addition, there was an improvement in the concentration index across the different vaccine antigens between UDHS 2000 and that of 2016, with the highest improvement registered for DPT3 from 0.85 to 0.95 respectively.

Decomposition of the inequity for UDHS 2016 showed that the region where the household resides (19.1%) and maternal education level (11.5%) were major contributors as to whether a child is fully immunized for their age. On the other hand, decomposition of the concentration indices for UDHS 2000 showed that the region where the household resides (47.5%), maternal education level (14.7%) and urban/rural household setting type (12.9%) were major contributors as to whether a child is fully immunized for their age.

Settings (urban/rural; 0%), sex of the child (1.1%), health insurance coverage (1.4%) and socioeconomic status (0.1%) had a limited influence. On the other hand, unexplained variation increased between 2000 to 2016 accounting for 22.9% and 67.5% of the variation in full immunization status respectively, indicating either random noise or the presence of factors not captured in the model.

All the regions of Uganda had an achievement level for full vaccination below 50%. The regions of Kigezi (47%), Acholi (45%), and Karamoja (43%) had the highest level of coverage, whereas North Buganda, Lango, and Tooro had the lowest rates, all at 29%. South Buganda shortly follows with 30% coverage. Based on the Wagstaff concentration index, the coverage for full immunization status was most equitable in Kigezi, Lango, and Karamoja, and least equitable in the regions of Bugisu and South Buganda.

*MCV1 coverage*

Generally, the coverage for the MCV1 increased from 68% (UDHS 2000) to 82.7% (UDHS 2016). The national estimate for MCV1 coverage (82.7%) was lower than that of DPT1 (93%), but higher than DPT3 (77%). The MCV1 coverage equity indices improved from 0.91 (UDHS 2000) to 0.95 (UDHS 2016). However, the MCV1 coverage equity indices were similar for the UDHS rounds of 2006, 2011 and 2016. The level of equity for MCV1 is also worse than for DPT1, but similar to DPT3 with Wagstaff and Erreyger concentration indices at 0.055 and 0.133, respectively, and an AEG of 0.180, signifying that 20% of the most disadvantaged people would need to increase MCV1 vaccine coverage by 18% to have similar levels to the top 20% most privileged. Level of maternal education (45.3%) and region (29.9%) were the biggest contributors of variation in equity for the MCV1 in the UDHS 2016. Similarly, region (43%), level of maternal education (26.7%) and urban/rural location for the households (15.7%) contributed most to the variation in equity for the MCV1 in the UDHS 2000.

The highest performing regions for MCV1 coverage were Kigezi (97%), Karamoja (93%), Tooro (89%), and Acholi (89%). Regions with the lowest coverage included Busoga at 76%, North Buganda at 77%, and South Buganda and Lango both at 78%. Wagstaff’s CI method found Karamoja (-0.008) and Bugisu (0.006) as experiencing the most equitable distribution of all regions in MCV1 vaccination status. South Buganda (0.076), North Buganda (0.064), and Acholi (0.063) displayed the lowest level of equity.

The selected VERSE parameters (detailed before the methods section) seem to explain well inequity in DPT3 and MCV1 vaccination status as the unexplained variation was found to be contributing only 33.9% and 5.1% (respectively) to the variation.

When computing the (more traditional) concentration index based on wealth only, it returned positive, or “pro-rich”, values for all vaccines and statuses in the earliest UDHS in 2000, and negative, or “pro-poor”, values afterward. This indicator reveals that the distribution of vaccines and their associated outcomes in Uganda benefited most wealthier people in 2000, and that this trend was reversed in the following years: now poorer Ugandans benefit most (although only slightly more) from it. While it displays an encouraging trend, using such wealth-based indicators may skew our assessment of distributional equity: adding the other demographical, social, and economic factors through the VERSE Equity Toolkit (detailed in the Methods) brings the concentration indices back in positive values, highlighting the adverse effect of compounding disadvantages on coverage.

**Figure S1: Uganda EPI schedule (2019).**


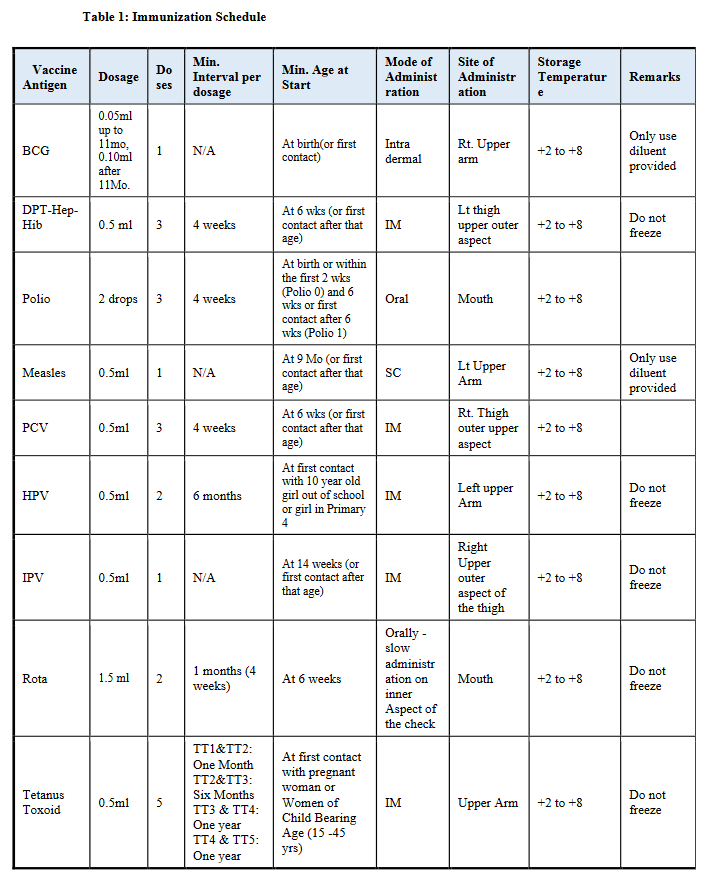


Source: UNEPI <https://www.health.go.ug/wp-content/uploads/2019/11/UNEPI-Immunization-guidelines.pdf>

Note that PCV was introduced in 2013 in Uganda and the rotavirus vaccine in 2018.

**National-level data**

**Levels of coverage and equity**

For the VERSE Equity Toolkit, “coverage” (Variable: *Coverage Results*) is a **measure of effectiveness**. In this analysis, coverage refers to vaccine coverage. When the issue of interest is instead a health outcome, this variable represents prevalence, defined as the proportion of the population impacted by the health outcome. For instance, Table S1 shows a 2.2% coverage for ZERO; it means that 2.2% of the children did not receive any vaccine and are considered “zero-dose children”.

The concentration index (CI) and the absolute equity gap (AEG) are **measures of equity**. A *greater* concentration index (with or without the Erreyger correction) indicates that the vaccine (or health outcome) coverage is *less* equitable (regardless of its level). AEG can be interpreted as the deviation in vaccination outcomes between the top and bottom quintiles of the population as ranked by unfair disadvantage such that a *greater* AEG value indicates *less* equity. The lower (“Low”) and upper (“High”) boundaries of the 95% confidence interval are displayed in the tables below.

**Table S1: National-level coverage data for 2016.**

| **Vaccine or health outcome ^1^** | **Coverage** | **Concentration index** | | | | | | | | | **Absolute Equity Gap**  *Multivariate* | | |
| --- | --- | --- | --- | --- | --- | --- | --- | --- | --- | --- | --- | --- | --- |
|  |  | *Wealth only (Wagstaff)* ^2^ | | | *Multivariate (Wagstaff)* | | | *Multivariate (Erreyger)* ^3^ | | |  |  |  |
|  |  | Base | Low | High | Base | Low | High | Base | Low | High | Base | Low | High |
| BCG | 94.2% | -0.071 | -0.084 | -0.058 | 0.020 | 0.017 | 0.023 | 0.077 | 0.074 | 0.080 | 0.098 | 0.082 | 0.114 |
| DPT1 | 92.7% | -0.062 | -0.075 | -0.049 | 0.020 | 0.016 | 0.024 | 0.071 | 0.067 | 0.075 | 0.106 | 0.084 | 0.128 |
| DPT2 | 86.8% | -0.052 | -0.064 | -0.040 | 0.033 | 0.027 | 0.039 | 0.106 | 0.100 | 0.112 | 0.146 | 0.121 | 0.171 |
| DPT3 | 76.8% | -0.042 | -0.053 | -0.031 | 0.054 | 0.046 | 0.062 | 0.150 | 0.142 | 0.158 | 0.183 | 0.154 | 0.212 |
| POLIO1 | 89.7% | -0.058 | -0.070 | -0.046 | 0.023 | 0.018 | 0.028 | 0.077 | 0.072 | 0.082 | 0.107 | 0.083 | 0.131 |
| POLIO2 | 82.5% | -0.051 | -0.063 | -0.039 | 0.039 | 0.032 | 0.046 | 0.120 | 0.113 | 0.127 | 0.153 | 0.126 | 0.180 |
| POLIO3 | 65.9% | -0.048 | -0.059 | -0.037 | 0.071 | 0.061 | 0.081 | 0.169 | 0.159 | 0.179 | 0.185 | 0.154 | 0.216 |
| PCV1 | 81.4% | -0.049 | -0.061 | -0.037 | 0.044 | 0.038 | 0.050 | 0.137 | 0.131 | 0.143 | 0.177 | 0.150 | 0.204 |
| PCV2 | 74.6% | -0.043 | -0.054 | -0.032 | 0.058 | 0.050 | 0.066 | 0.161 | 0.153 | 0.169 | 0.215 | 0.186 | 0.244 |
| PCV3 | 64.1% | -0.035 | -0.046 | -0.024 | 0.071 | 0.061 | 0.081 | 0.164 | 0.154 | 0.174 | 0.197 | 0.166 | 0.228 |
| MCV1 | 82.7% | -0.029 | -0.040 | -0.018 | 0.055 | 0.045 | 0.065 | 0.133 | 0.123 | 0.143 | 0.180 | 0.149 | 0.211 |
| ZERO | 2.2% | -0.078 | -0.091 | -0.065 | 0.425 | 0.341 | 0.509 | 0.022 | -0.062 | 0.106 | 0.027 | 0.019 | 0.035 |
| FULL | 40.8% | -0.043 | -0.054 | -0.032 | 0.110 | 0.096 | 0.124 | 0.181 | 0.167 | 0.195 | 0.230 | 0.199 | 0.261 |
| COMPLETE | 39.0% | -0.044 | -0.063 | -0.025 | 0.140 | 0.114 | 0.166 | 0.218 | 0.192 | 0.244 | 0.267 | 0.214 | 0.320 |

Notes: ^1.^ ZERO, the child didn’t receive any vaccine by 12 months old; FULL, the child is under 24 months old and is fully immunized for their age; COMPLETE, the child is above 24 months and completed the routine pediatric immunization schedule.
^2.^ Concentration index based on households ranked by socioeconomic status (as defined in the DHS) only.
^3.^ See (1, 2) for details on the Erreyger’s correction.

**Table S2: National-level coverage data for 2011.**

| **Vaccine or health outcome ^1^** | **Coverage** | **Concentration index** | | | | | | | | | **Absolute Equity Gap**  *Multivariate* | | |
| --- | --- | --- | --- | --- | --- | --- | --- | --- | --- | --- | --- | --- | --- |
|  |  | *Wealth only (Wagstaff)* ^2^ | | | *Multivariate (Wagstaff)* | | | *Multivariate (Erreyger)* ^3^ | | |  |  |  |
|  |  | Base | Low | High | Base | Low | High | Base | Low | High | Base | Low | High |
| BCG | 92.1% | -0.079 | -0.083 | -0.075 | 0.029 | 0.025 | 0.033 | 0.106 | 0.102 | 0.110 | 0.141 | 0.121 | 0.161 |
| DPT1 | 89.0% | -0.074 | -0.079 | -0.069 | 0.029 | 0.024 | 0.034 | 0.104 | 0.099 | 0.109 | 0.147 | 0.123 | 0.171 |
| DPT2 | 80.8% | -0.063 | -0.068 | -0.058 | 0.047 | 0.040 | 0.054 | 0.152 | 0.145 | 0.159 | 0.181 | 0.154 | 0.208 |
| DPT3 | 66.9% | -0.047 | -0.053 | -0.041 | 0.078 | 0.069 | 0.087 | 0.207 | 0.198 | 0.216 | 0.263 | 0.230 | 0.296 |
| POLIO1 | 89.0% | -0.073 | -0.078 | -0.068 | 0.025 | 0.020 | 0.030 | 0.089 | 0.084 | 0.094 | 0.120 | 0.096 | 0.144 |
| POLIO2 | 79.5% | -0.063 | -0.068 | -0.058 | 0.041 | 0.034 | 0.048 | 0.129 | 0.122 | 0.136 | 0.165 | 0.136 | 0.194 |
| POLIO3 | 57.3% | -0.039 | -0.045 | -0.033 | 0.076 | 0.064 | 0.088 | 0.169 | 0.157 | 0.181 | 0.221 | 0.186 | 0.256 |
| MCV1 | 70.9% | -0.043 | -0.048 | -0.038 | 0.050 | 0.041 | 0.059 | 0.137 | 0.128 | 0.146 | 0.157 | 0.124 | 0.190 |
| ZERO | 0.8% | -0.092 | -0.096 | -0.088 | 0.527 | 0.424 | 0.630 | 0.016 | -0.087 | 0.119 | 0.019 | 0.011 | 0.027 |
| FULL | 48.9% | -0.032 | -0.043 | -0.021 | 0.111 | 0.087 | 0.135 | 0.216 | 0.192 | 0.240 | 0.321 | 0.258 | 0.384 |
| COMPLETE | 54.8% | -0.027 | -0.040 | -0.014 | 0.144 | 0.114 | 0.174 | 0.305 | 0.275 | 0.335 | 0.379 | 0.297 | 0.461 |

**Table S3: National-level coverage data for 2006.**

| **Vaccine or health outcome ^1^** | **Coverage** | **Concentration index** | | | | | | | | | **Absolute Equity Gap**  *Multivariate* | | |
| --- | --- | --- | --- | --- | --- | --- | --- | --- | --- | --- | --- | --- | --- |
|  |  | *Wealth only (Wagstaff)* ^2^ | | | *Multivariate (Wagstaff)* | | | *Multivariate (Erreyger)* ^3^ | | |  |  |  |
|  |  | Base | Low | High | Base | Low | High | Base | Low | High | Base | Low | High |
| BCG | 88.7% | -0.088 | -0.090 | -0.086 | 0.038 | 0.033 | 0.043 | 0.135 | 0.130 | 0.140 | 0.162 | 0.138 | 0.186 |
| DPT1 | 41.2% | -0.060 | -0.064 | -0.056 | 0.087 | 0.071 | 0.103 | 0.143 | 0.127 | 0.159 | 0.185 | 0.152 | 0.218 |
| DPT2 | 35.5% | -0.063 | -0.067 | -0.059 | 0.110 | 0.093 | 0.127 | 0.153 | 0.136 | 0.170 | 0.196 | 0.165 | 0.227 |
| DPT3 | 24.5% | -0.071 | -0.075 | -0.067 | 0.147 | 0.125 | 0.169 | 0.143 | 0.121 | 0.165 | 0.159 | 0.130 | 0.188 |
| POLIO1 | 87.5% | -0.084 | -0.086 | -0.082 | 0.031 | 0.026 | 0.036 | 0.107 | 0.102 | 0.112 | 0.123 | 0.098 | 0.148 |
| POLIO2 | 76.9% | -0.064 | -0.067 | -0.061 | 0.041 | 0.033 | 0.049 | 0.122 | 0.114 | 0.130 | 0.172 | 0.141 | 0.203 |
| POLIO3 | 53.8% | -0.044 | -0.047 | -0.041 | 0.063 | 0.050 | 0.076 | 0.130 | 0.117 | 0.143 | 0.169 | 0.136 | 0.202 |
| MCV1 | 68.4% | -0.053 | -0.056 | -0.050 | 0.059 | 0.049 | 0.069 | 0.150 | 0.140 | 0.160 | 0.177 | 0.144 | 0.210 |
| ZERO | 1.6% | -0.100 | -0.101 | -0.099 | 0.473 | 0.394 | 0.552 | 0.027 | -0.052 | 0.106 | 0.032 | 0.022 | 0.042 |
| FULL | 12.5% | -0.070 | -0.070 | -0.070 | 0.180 | 0.123 | 0.237 | 0.090 | 0.033 | 0.147 | 0.117 | 0.076 | 0.158 |
| COMPLETE | 15.8% | -0.059 | -0.062 | -0.056 | 0.189 | 0.122 | 0.256 | 0.125 | 0.058 | 0.192 | 0.167 | 0.104 | 0.230 |

Notes: ^1.^ ZERO, the child didn’t receive any vaccine by 12 months old; FULL, the child is under 24 months old and is fully immunized for their age; COMPLETE, the child is above 24 months and completed the routine pediatric immunization schedule.
^2.^ Concentration index based on households ranked by socioeconomic status (as defined in the DHS) only.
^3.^ See (1, 2) for details on the Erreyger’s correction.

**Table S4: National-level coverage data for 2000.**

| **Vaccine or health outcome ^1^** | **Coverage** | **Concentration index** | | | | | | | | | **Absolute Equity Gap**  *Multivariate* | | |
| --- | --- | --- | --- | --- | --- | --- | --- | --- | --- | --- | --- | --- | --- |
|  |  | *Wealth only (Wagstaff)* ^2^ | | | *Multivariate (Wagstaff)* | | | *Multivariate (Erreyger)* ^3^ | | |  |  |  |
|  |  | Base | Low | High | Base | Low | High | Base | Low | High | Base | Low | High |
| BCG | 77.4% | 0.167 | 0.162 | 0.172 | 0.072 | 0.064 | 0.080 | 0.226 | 0.218 | 0.234 | 0.269 | 0.240 | 0.298 |
| DPT1 | 76.8% | 0.157 | 0.152 | 0.162 | 0.070 | 0.062 | 0.078 | 0.211 | 0.203 | 0.219 | 0.234 | 0.203 | 0.265 |
| DPT2 | 65.6% | 0.135 | 0.129 | 0.141 | 0.101 | 0.090 | 0.112 | 0.260 | 0.249 | 0.271 | 0.305 | 0.270 | 0.340 |
| DPT3 | 49.7% | 0.127 | 0.120 | 0.134 | 0.150 | 0.136 | 0.164 | 0.295 | 0.281 | 0.309 | 0.358 | 0.323 | 0.393 |
| POLIO1 | 83.0% | 0.173 | 0.169 | 0.177 | 0.051 | 0.044 | 0.058 | 0.166 | 0.159 | 0.173 | 0.185 | 0.156 | 0.214 |
| POLIO2 | 73.5% | 0.144 | 0.140 | 0.148 | 0.073 | 0.064 | 0.082 | 0.209 | 0.200 | 0.218 | 0.261 | 0.228 | 0.294 |
| POLIO3 | 56.0% | 0.117 | 0.112 | 0.122 | 0.120 | 0.107 | 0.133 | 0.257 | 0.244 | 0.270 | 0.317 | 0.282 | 0.352 |
| MCV1 | 67.8% | 0.123 | 0.117 | 0.129 | 0.087 | 0.075 | 0.099 | 0.200 | 0.188 | 0.212 | 0.234 | 0.197 | 0.271 |
| ZERO | 8.4% | 0.236 | 0.233 | 0.239 | 0.427 | 0.379 | 0.475 | 0.106 | 0.058 | 0.154 | 0.123 | 0.103 | 0.143 |
| FULL | 38.1% | 0.133 | 0.127 | 0.139 | 0.163 | 0.145 | 0.181 | 0.260 | 0.242 | 0.278 | 0.291 | 0.254 | 0.328 |
| COMPLETE | 47.9% | 0.133 | 0.124 | 0.142 | 0.154 | 0.134 | 0.174 | 0.309 | 0.289 | 0.329 | 0.363 | 0.312 | 0.414 |

Notes: ^1.^ ZERO, the child didn’t receive any vaccine by 12 months old; FULL, the child is under 24 months old and is fully immunized for their age; COMPLETE, the child is above 24 months and completed the routine pediatric immunization schedule.
^2.^ Concentration index based on households ranked by socioeconomic status (as defined in the DHS) only.
^3.^ See (1, 2) for details on the Erreyger’s correction.

**Figure S2: Vaccine coverage and equity maps for 2011.**


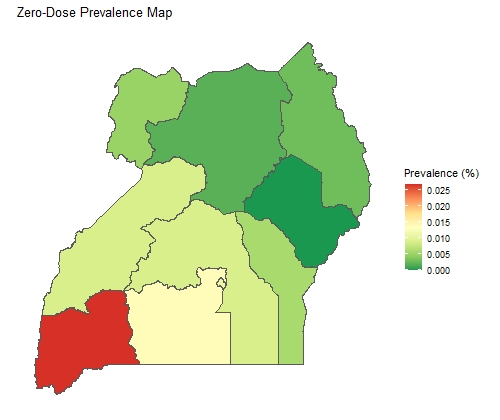


**A**


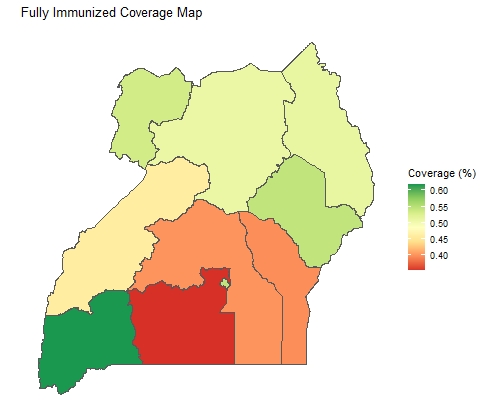


**C**


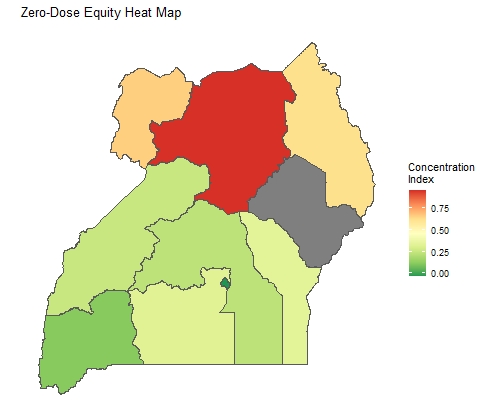


**B**


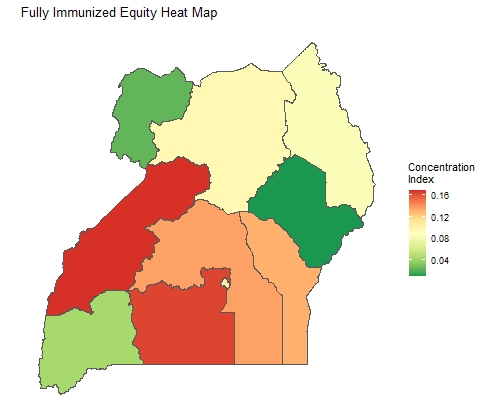


**D**


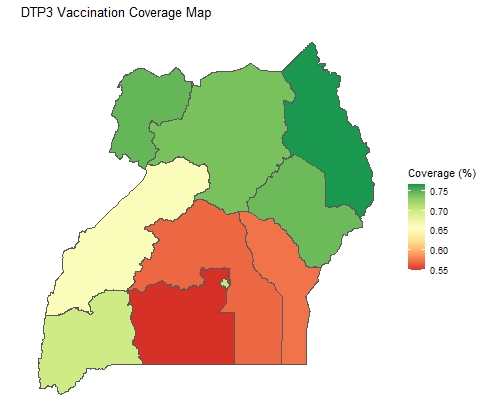


**E**


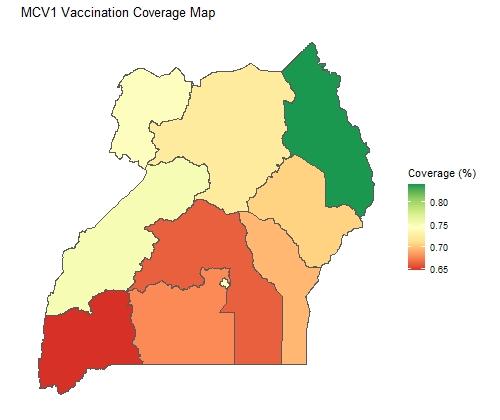


**G**


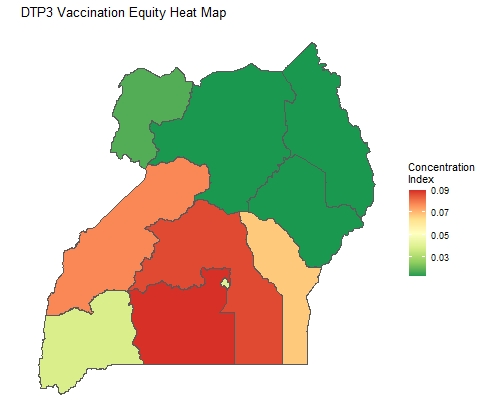


**F**


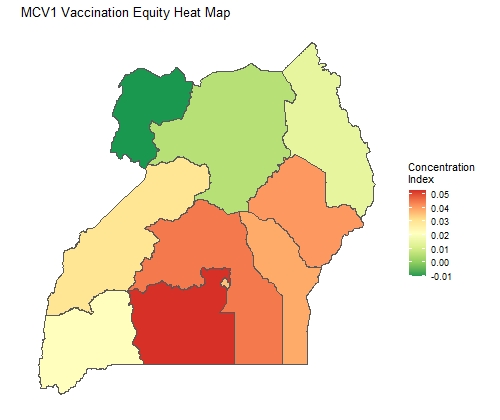


**H**

**Figure S3: Vaccine coverage and equity maps for 2006.**


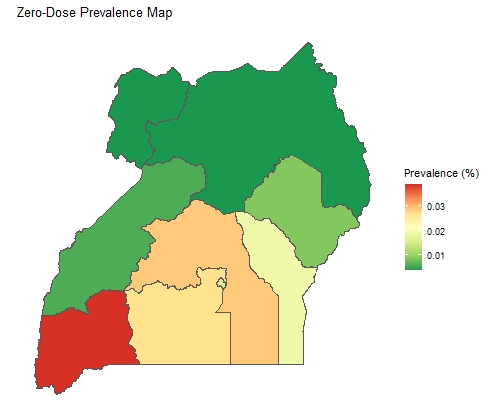


**A**


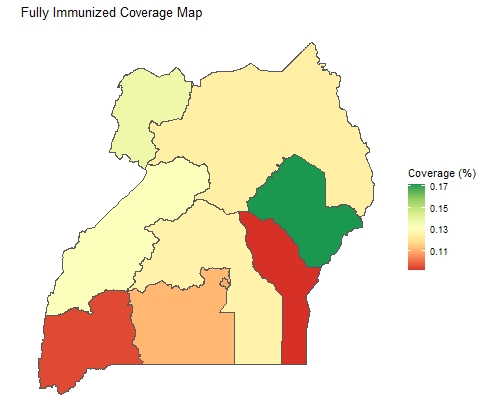


**C**


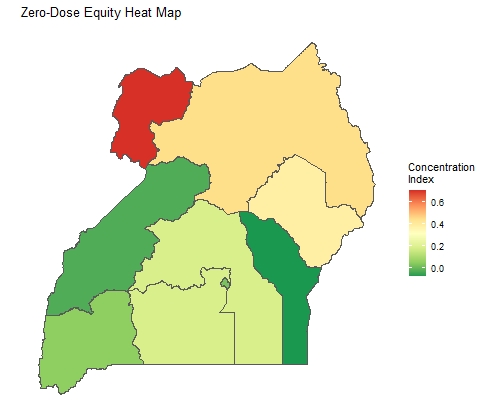


**B**


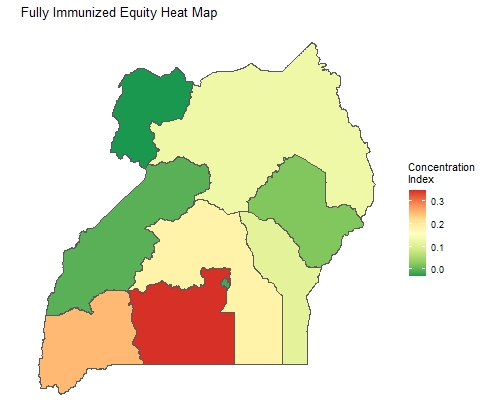


**D**


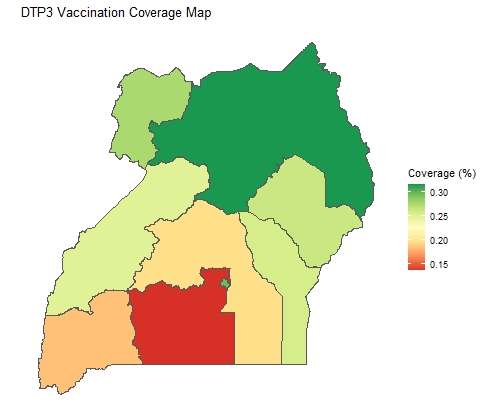


**E**


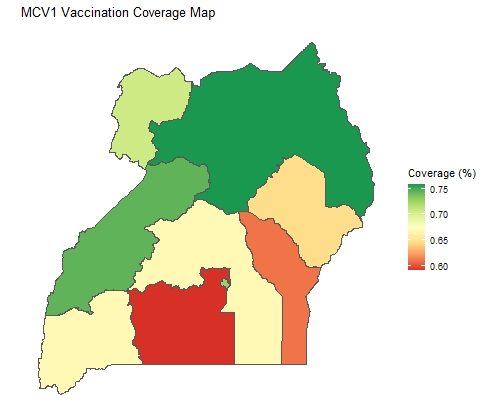


**G**


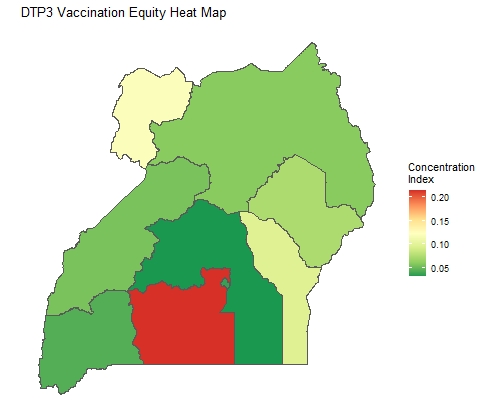


**F**


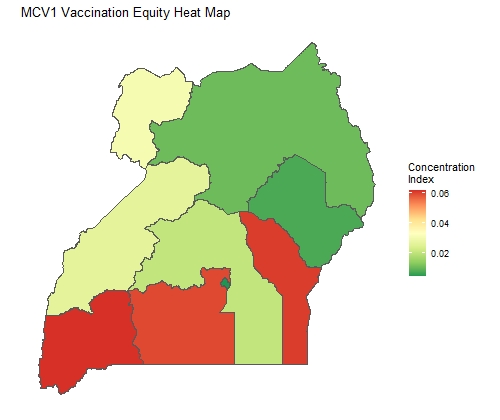


**H**

**Figure S4: Vaccine coverage and equity maps for 2000.**


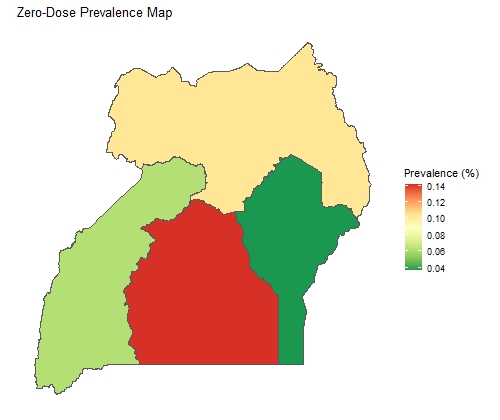


**A**


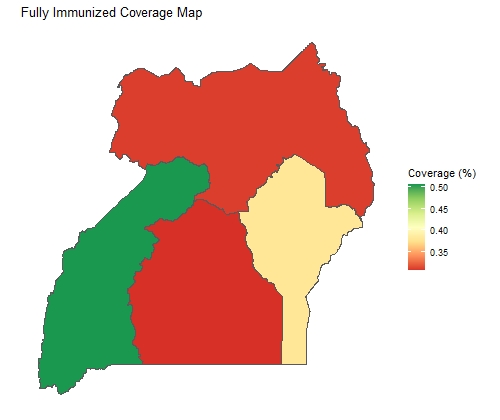


**C**


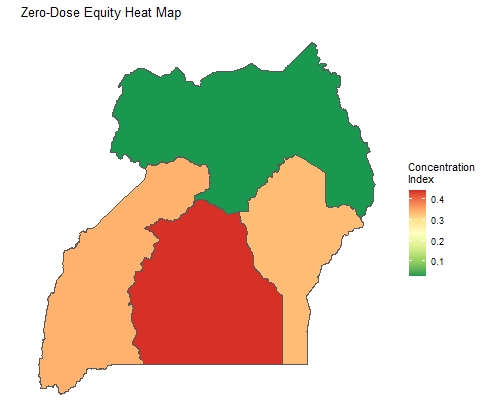


**B**


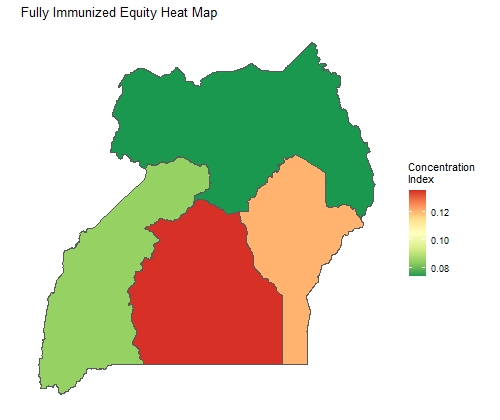


**D**


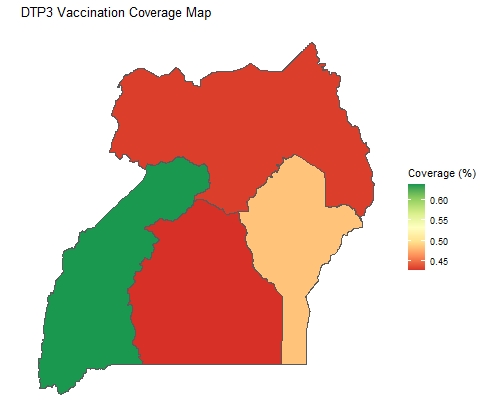


**E**


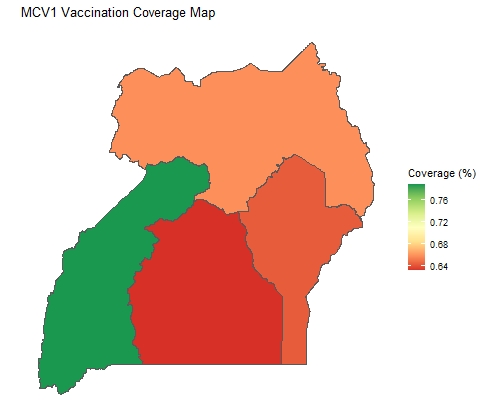


**G**


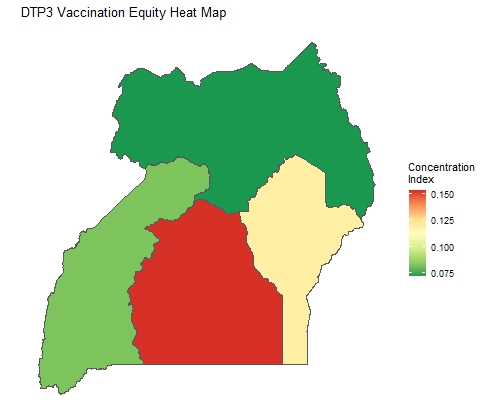


**F**


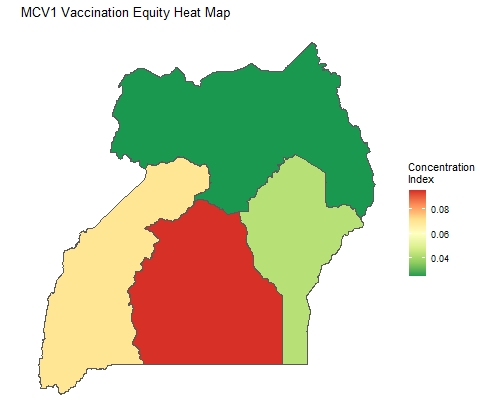


**H**

**Figure S5: Effect of fair and unfair factors on zero-dose prevalence from 2000 to 2016.**


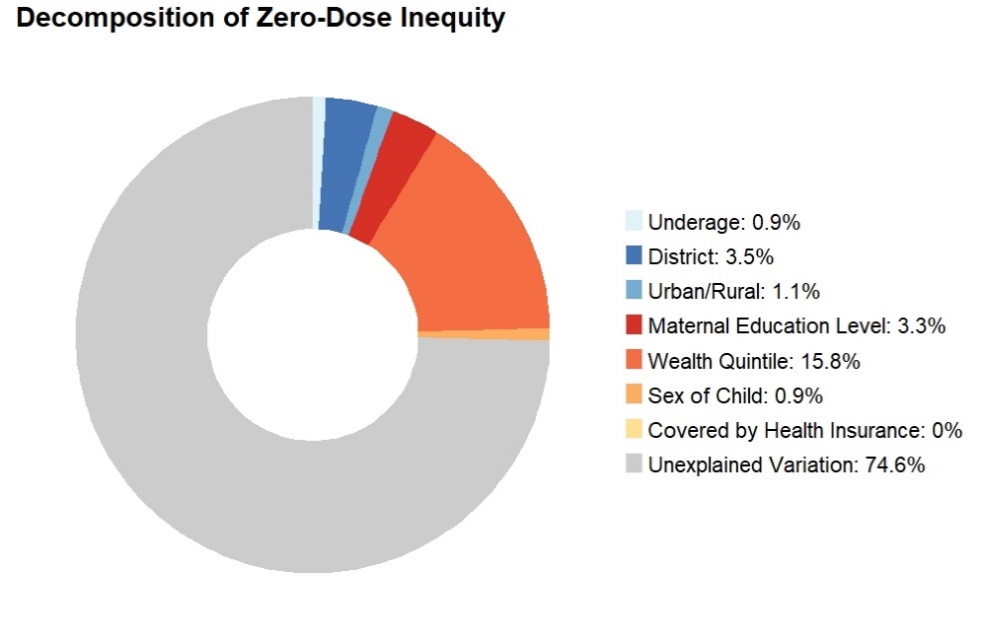


**A**

**B**


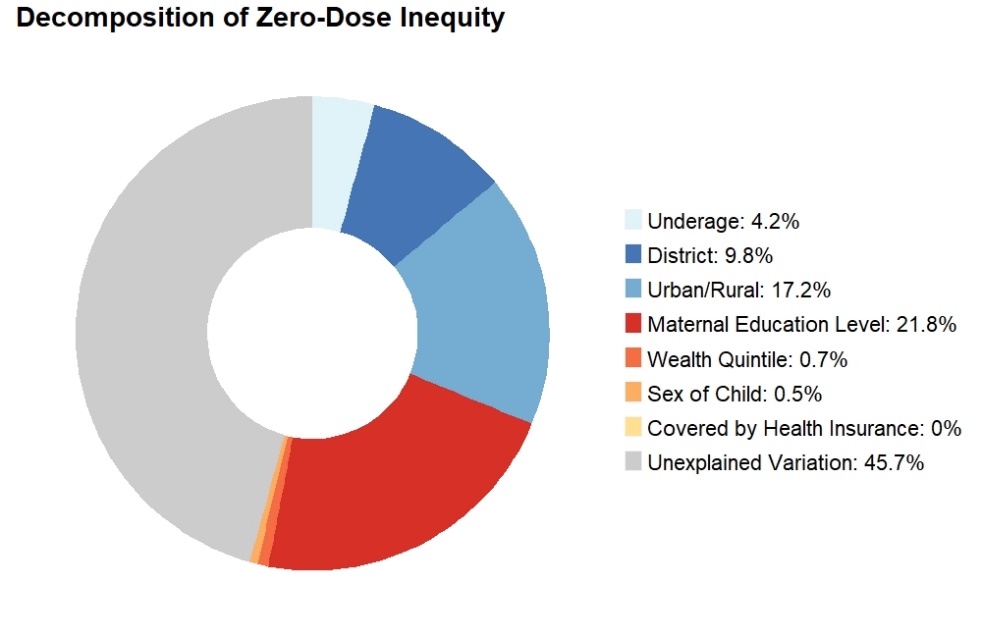


**C**


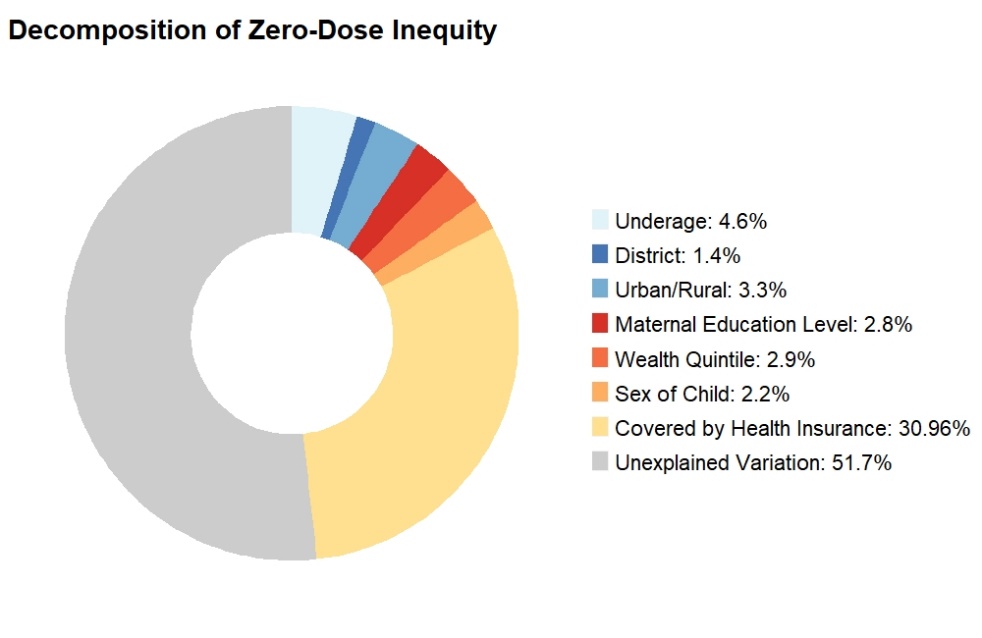


2000

2011

2006

Notes: Being underaged for vaccination (a *fair* contributor) refers to children who are younger than 12 months old and have not received any vaccines yet: they are at risk of becoming “zero-dose” as per Gavi’s definition.

**Figure S6: Effect of fair and unfair factors on being fully immunized for age from 2000 to 2016.**


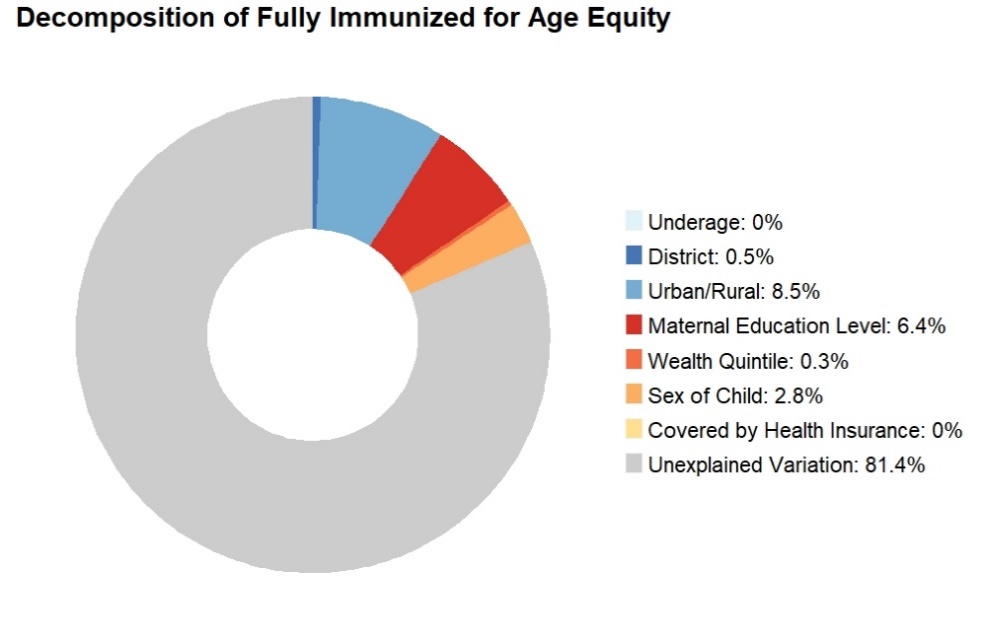


**A**

**B**


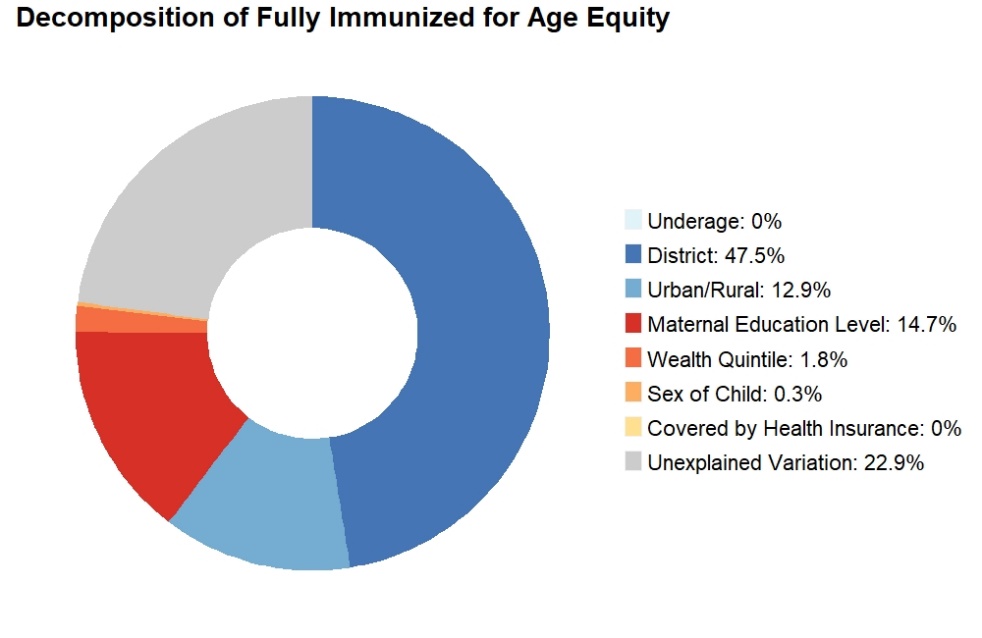


**C**


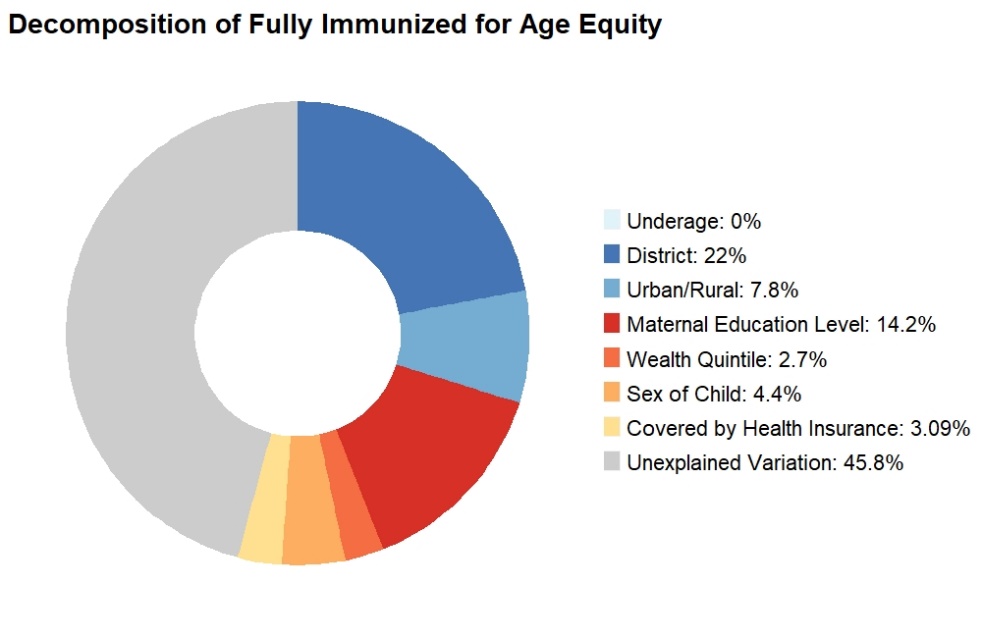


2000

2011

2006

**Figure S7: Effect of fair and unfair factors of DPT3 vaccine coverage from 2000 to 2016.**


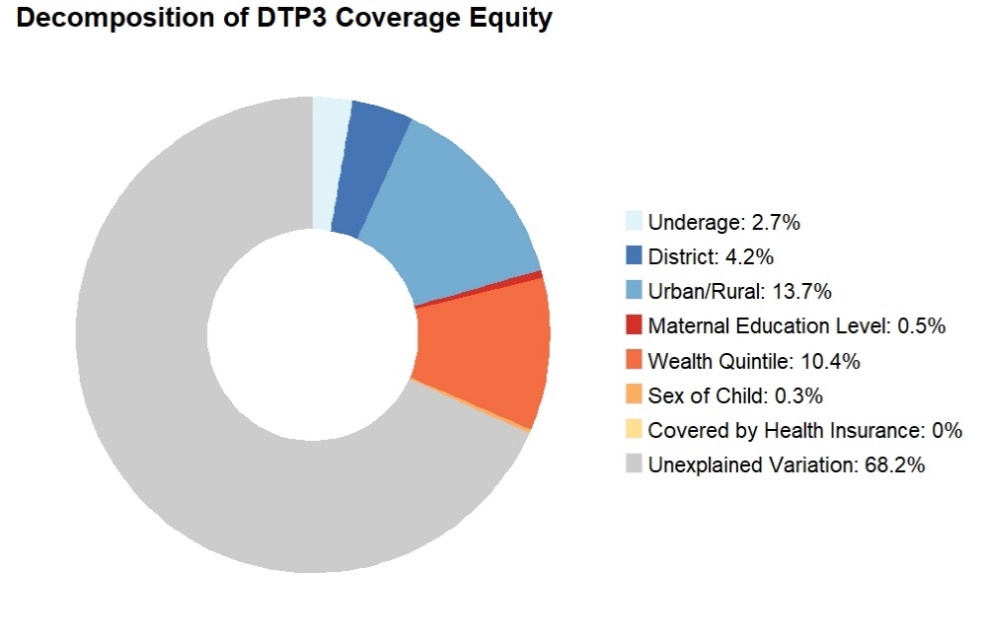


**A**

**B**


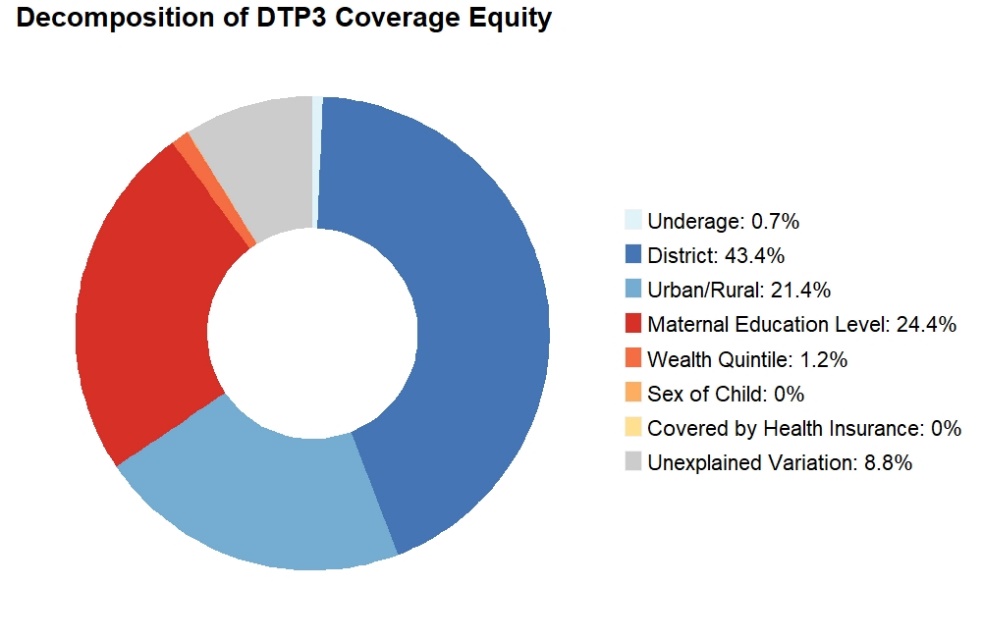


**C**


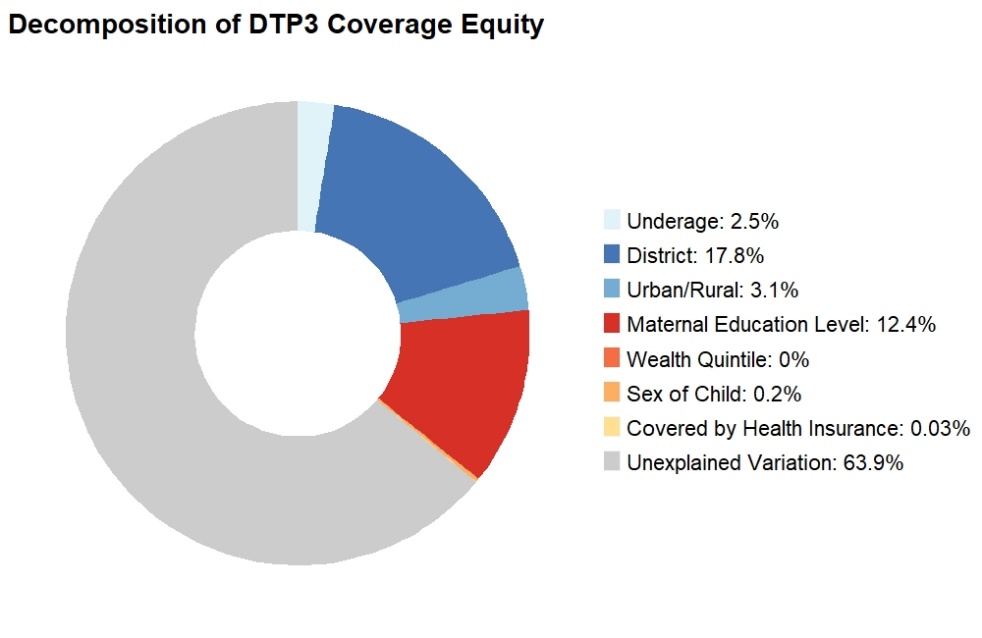


2000

2011

2006

**Figure S8: Effect of fair and unfair factors on zero-dose prevalence from 2000 to 2016.**


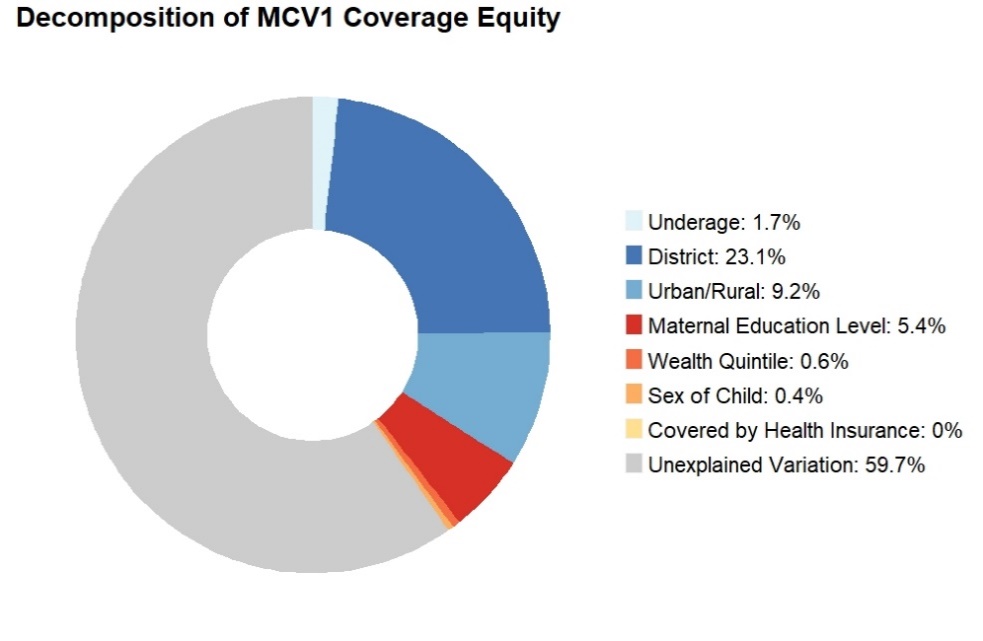


**A**

**B**


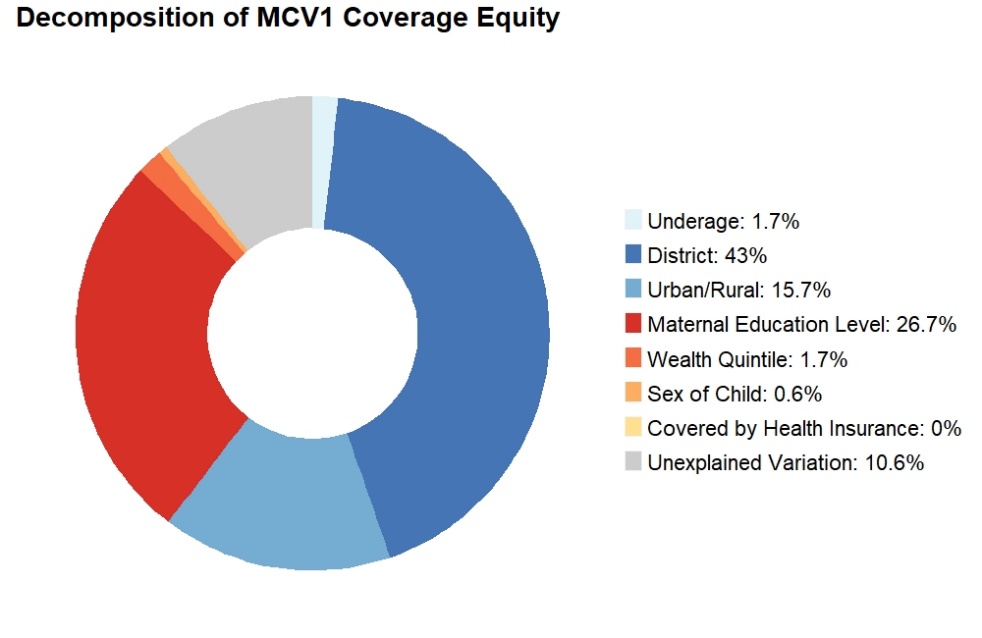


**C**


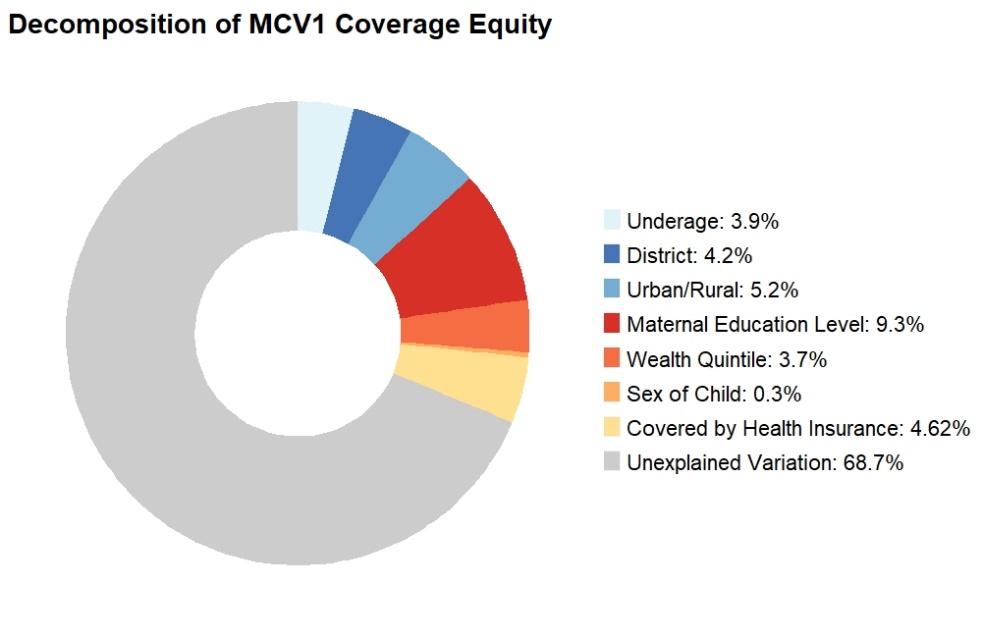


2000

2011

2006

**Subnational data**

**Table S5: Subnational vaccine coverage rates for 2016.**

| **Region** | **Vaccines and health outcomes** | | | | | | | | | | | | | |
| --- | --- | --- | --- | --- | --- | --- | --- | --- | --- | --- | --- | --- | --- | --- |
|  | **BCG** | **DPT1** | **DPT2** | **DPT3** | **OPV1** | **OPV2** | **OPV3** | **PCV1** | **PCV2** | **PCV3** | **MCV1** | **ZERO** | **FULL** | **COMP** |
| Kampala | 96.8% | 93.1% | 86.0% | 78.3% | 90.8% | 79.3% | 56.9% | 88.4% | 79.0% | 67.9% | 85.3% | 0.7% | 39.5% | 34.8% |
| South Buganda | 90.0% | 88.2% | 81.1% | 70.9% | 86.5% | 76.8% | 56.4% | 76.6% | 68.1% | 58.6% | 77.5% | 4.8% | 35.5% | 30.9% |
| North Buganda | 90.5% | 89.2% | 83.6% | 72.1% | 85.2% | 78.1% | 58.1% | 77.7% | 69.2% | 57.8% | 76.5% | 3.5% | 33.4% | 33.8% |
| Busoga | 93.4% | 90.8% | 87.3% | 73.1% | 88.2% | 82.2% | 62.5% | 81.3% | 75.7% | 66.5% | 76.0% | 3.0% | 38.8% | 41.8% |
| Bukedi | 96.9% | 96.6% | 91.6% | 79.8% | 94.2% | 83.8% | 63.7% | 89.6% | 85.1% | 69.3% | 80.6% | 1.1% | 43.5% | 43.0% |
| Bugisu | 97.4% | 95.6% | 80.1% | 69.7% | 82.9% | 70.8% | 59.4% | 87.1% | 69.4% | 54.8% | 81.4% | 0.0% | 36.6% | 26.6% |
| Teso | 97.6% | 96.0% | 94.0% | 89.3% | 93.9% | 92.3% | 80.3% | 79.2% | 74.6% | 67.0% | 86.9% | 0.7% | 44.0% | 39.0% |
| Karamoja | 98.0% | 96.7% | 89.8% | 84.5% | 93.1% | 86.9% | 69.7% | 91.5% | 85.0% | 76.6% | 93.0% | 0.6% | 52.3% | 53.9% |
| Lango | 96.1% | 91.9% | 87.8% | 77.5% | 87.9% | 81.8% | 65.0% | 68.5% | 64.3% | 54.8% | 78.4% | 1.6% | 32.7% | 24.8% |
| Acholi | 96.9% | 94.2% | 90.2% | 84.3% | 89.8% | 87.8% | 75.5% | 85.3% | 77.8% | 68.8% | 88.6% | 2.2% | 46.8% | 38.3% |
| West Nile | 95.2% | 94.1% | 89.4% | 81.2% | 92.3% | 87.5% | 75.4% | 86.6% | 82.3% | 71.5% | 85.3% | 2.5% | 51.6% | 52.1% |
| Bunyoro | 92.1% | 92.0% | 85.7% | 78.4% | 91.2% | 85.2% | 73.2% | 81.5% | 75.7% | 68.5% | 84.7% | 2.3% | 48.0% | 47.0% |
| Tooro | 95.5% | 92.2% | 84.0% | 71.9% | 90.2% | 81.3% | 64.7% | 74.9% | 66.0% | 56.8% | 88.9% | 1.2% | 34.1% | 36.6% |
| Ankole | 92.4% | 94.5% | 90.5% | 78.3% | 93.1% | 86.6% | 71.7% | 82.7% | 80.6% | 69.9% | 85.5% | 1.6% | 44.3% | 48.2% |
| Kigezi | 97.1% | 96.8% | 90.3% | 83.1% | 96.2% | 88.1% | 77.6% | 89.0% | 84.9% | 71.6% | 96.8% | 0.0% | 52.1% | 48.2% |

**Table S6: Subnational vaccine coverage rates for 2011.**

| **Region** | **Vaccines and health outcomes** | | | | | | | | | | |
| --- | --- | --- | --- | --- | --- | --- | --- | --- | --- | --- | --- |
|  | **BCG** | **DPT1** | **DPT2** | **DPT3** | **OPV1** | **OPV2** | **OPV3** | **MCV1** | **ZERO** | **FULL** | **COMP** |
| Kampala | 94.8% | 91.2% | 80.4% | 70.7% | 89.8% | 79.9% | 63.4% | 74.6% | 1.1% | 54.6% | 52.9% |
| Central 1 | 85.7% | 83.6% | 71.4% | 54.9% | 84.0% | 69.8% | 43.3% | 67.9% | 1.4% | 35.2% | 40.4% |
| Central 2 | 90.7% | 84.1% | 73.3% | 56.9% | 87.2% | 73.3% | 48.0% | 66.3% | 0.9% | 40.0% | 42.6% |
| East central | 92.6% | 88.9% | 77.1% | 57.4% | 88.7% | 78.6% | 57.9% | 69.6% | 0.6% | 39.7% | 47.2% |
| Eastern | 97.1% | 94.1% | 87.1% | 74.5% | 93.9% | 84.8% | 56.8% | 70.7% | 0.0% | 54.6% | 61.3% |
| North | 94.0% | 92.1% | 88.5% | 74.1% | 90.0% | 83.2% | 56.2% | 72.4% | 0.2% | 50.8% | 54.4% |
| Karamoja | 97.6% | 92.7% | 86.1% | 76.8% | 91.8% | 83.7% | 53.4% | 84.1% | 0.3% | 51.2% | 45.9% |
| West Nile | 96.3% | 92.8% | 86.1% | 74.8% | 92.4% | 84.7% | 60.2% | 74.6% | 0.5% | 53.5% | 61.7% |
| Western | 92.5% | 91.8% | 83.3% | 66.3% | 90.8% | 81.9% | 63.2% | 75.1% | 0.9% | 46.1% | 53.0% |
| Southwest | 83.8% | 80.8% | 76.8% | 70.0% | 81.9% | 76.9% | 67.4% | 64.8% | 2.7% | 62.0% | 76.0% |

**Table S7: Subnational vaccine coverage rates for 2006.**

| **Region** | **Vaccines and health outcomes** | | | | | | | | | | |
| --- | --- | --- | --- | --- | --- | --- | --- | --- | --- | --- | --- |
|  | **BCG** | **DPT1** | **DPT2** | **DPT3** | **OPV1** | **OPV2** | **OPV3** | **MCV1** | **ZERO** | **FULL** | **COMP** |
| Central 1 | 76.5% | 33.7% | 24.0% | 13.8% | 76.3% | 61.3% | 44.8% | 59.2% | 2.7% | 11.2% | 13.6% |
| Central 2 | 83.9% | 33.8% | 28.8% | 19.7% | 84.8% | 75.5% | 56.5% | 67.0% | 2.9% | 12.7% | 16.3% |
| Kampala | 90.8% | 42.7% | 39.5% | 29.7% | 87.9% | 79.4% | 53.2% | 72.8% | 1.6% | 11.0% | 11.8% |
| East central | 88.7% | 42.5% | 36.2% | 25.8% | 86.3% | 72.2% | 48.6% | 61.1% | 1.9% | 9.2% | 12.8% |
| Eastern | 91.4% | 42.7% | 37.9% | 26.3% | 89.6% | 78.6% | 55.3% | 64.7% | 0.9% | 17.2% | 22.3% |
| North | 94.0% | 48.2% | 41.9% | 31.6% | 91.7% | 80.6% | 50.9% | 75.9% | 0.4% | 12.5% | 17.6% |
| West Nile | 93.5% | 40.0% | 35.9% | 27.6% | 93.7% | 83.1% | 52.6% | 70.8% | 0.4% | 13.8% | 14.9% |
| Western | 93.2% | 46.3% | 40.6% | 25.1% | 91.3% | 81.3% | 59.9% | 74.5% | 0.6% | 13.3% | 13.5% |
| Southwest | 81.5% | 32.5% | 28.1% | 18.6% | 83.1% | 77.6% | 58.8% | 67.0% | 3.9% | 9.5% | 14.2% |

**Table S8: Subnational vaccine coverage rates for 2000.**

| **Region (2000)** | **Vaccines and health outcomes** | | | | | | | | | | |
| --- | --- | --- | --- | --- | --- | --- | --- | --- | --- | --- | --- |
|  | **BCG** | **DPT1** | **DPT2** | **DPT3** | **OPV1** | **OPV2** | **OPV3** | **MCV1** | **ZERO** | **FULL** | **COMP** |
| Central | 70.3% | 68.3% | 56.6% | 42.8% | 74.9% | 63.7% | 43.4% | 63.2% | 14.2% | 30.8% | 41.2% |
| Eastern | 83.5% | 80.3% | 66.9% | 48.6% | 87.8% | 75.8% | 59.5% | 64.3% | 3.7% | 38.2% | 44.6% |
| Northern | 75.9% | 76.5% | 62.4% | 43.2% | 80.3% | 73.6% | 53.2% | 65.9% | 10.3% | 31.2% | 37.5% |
| Western | 79.1% | 82.9% | 76.9% | 63.9% | 88.4% | 82.4% | 68.7% | 79.0% | 6.3% | 50.7% | 66.2% |

**Table S9: Subnational vaccine equity metric for 2016: Wagstaff multivariate concentration index.**

| **Region** | **Vaccines and health outcomes** | | | | | | | | | | | | | |
| --- | --- | --- | --- | --- | --- | --- | --- | --- | --- | --- | --- | --- | --- | --- |
|  | **BCG** | **DPT1** | **DPT2** | **DPT3** | **OPV1** | **OPV2** | **OPV3** | **PCV1** | **PCV2** | **PCV3** | **MCV1** | **ZERO** | **FULL** | **COMP** |
| Kampala | 0.008 | 0.012 | 0.036 | 0.066 | 0.008 | 0.043 | 0.063 | 0.019 | 0.050 | 0.059 | 0.036 | 0.229 | 0.057 | 0.173 |
| South Buganda | 0.030 | 0.027 | 0.042 | 0.059 | 0.025 | 0.032 | 0.067 | 0.053 | 0.063 | 0.080 | 0.076 | 0.312 | 0.140 | 0.146 |
| North Buganda | 0.028 | 0.029 | 0.040 | 0.059 | 0.030 | 0.049 | 0.012 | 0.051 | 0.088 | 0.117 | 0.064 | 0.254 | 0.089 | 0.119 |
| Busoga | 0.009 | 0.018 | 0.017 | 0.048 | 0.006 | 0.023 | 0.033 | 0.005 | 0.024 | 0.066 | 0.033 | -0.142 | 0.052 | -0.002 |
| Bukedi | 0.008 | 0.002 | 0.029 | 0.032 | 0.000 | 0.028 | 0.047 | -0.006 | 0.026 | 0.037 | 0.045 | 0.397 | 0.063 | 0.059 |
| Bugisu | 0.005 | 0.017 | 0.027 | 0.022 | 0.014 | 0.037 | 0.042 | 0.033 | 0.029 | 0.036 | 0.006 | NA | 0.111 | 0.211 |
| Teso | -0.001 | -0.013 | -0.005 | -0.010 | -0.004 | 0.004 | 0.004 | 0.007 | 0.014 | 0.020 | 0.022 | -0.055 | 0.046 | 0.054 |
| Karamoja | 0.005 | 0.004 | -0.007 | -0.017 | 0.004 | 0.004 | -0.026 | 0.019 | 0.030 | 0.008 | -0.008 | 0.029 | 0.040 | 0.031 |
| Lango | 0.005 | 0.007 | 0.019 | 0.028 | 0.005 | 0.022 | 0.007 | 0.007 | 0.024 | 0.014 | 0.033 | 0.182 | 0.025 | -0.136 |
| Acholi | 0.000 | 0.019 | 0.023 | 0.017 | 0.021 | 0.022 | 0.024 | 0.042 | 0.017 | 0.045 | 0.063 | 0.284 | 0.072 | 0.078 |
| West Nile | 0.013 | 0.008 | 0.012 | 0.024 | 0.008 | 0.018 | 0.046 | 0.001 | 0.009 | 0.024 | 0.017 | 0.307 | 0.076 | 0.097 |
| Bunyoro | 0.025 | 0.020 | 0.027 | 0.044 | 0.016 | 0.016 | 0.031 | 0.042 | 0.031 | 0.050 | 0.030 | 0.553 | 0.048 | 0.027 |
| Tooro | 0.006 | 0.015 | 0.031 | 0.024 | 0.015 | 0.026 | 0.006 | 0.017 | 0.023 | 0.026 | 0.032 | 0.000 | 0.047 | 0.044 |
| Ankole | 0.023 | 0.022 | 0.027 | 0.040 | 0.023 | 0.025 | 0.054 | 0.022 | 0.038 | 0.033 | 0.027 | 0.257 | 0.059 | 0.139 |
| Kigezi | 0.017 | 0.011 | 0.032 | 0.031 | 0.015 | 0.015 | 0.020 | 0.024 | 0.032 | 0.037 | 0.055 | NA | -0.007 | 0.095 |

**Table S10: Subnational vaccine equity metric for 2011: Wagstaff multivariate concentration index.**

| **Region** | **Vaccines and health outcomes** | | | | | | | | | | |
| --- | --- | --- | --- | --- | --- | --- | --- | --- | --- | --- | --- |
|  | **BCG** | **DPT1** | **DPT2** | **DPT3** | **OPV1** | **OPV2** | **OPV3** | **MCV1** | **ZERO** | **FULL** | **COMP** |
| Kampala | 0.008 | 0.006 | 0.017 | 0.041 | 0.015 | 0.043 | 0.033 | 0.036 | -0.032 | 0.105 | 0.012 |
| Central 1 | 0.042 | 0.033 | 0.058 | 0.090 | 0.015 | 0.037 | 0.064 | 0.053 | 0.327 | 0.165 | 0.164 |
| Central 2 | 0.021 | 0.024 | 0.040 | 0.087 | 0.018 | 0.038 | 0.079 | 0.045 | 0.231 | 0.137 | 0.171 |
| East central | 0.010 | 0.004 | 0.027 | 0.068 | 0.001 | 0.017 | 0.032 | 0.039 | 0.341 | 0.132 | 0.093 |
| Eastern | 0.002 | 0.012 | 0.012 | 0.013 | 0.009 | 0.021 | 0.004 | 0.041 | NA | 0.009 | -0.019 |
| North | 0.014 | 0.006 | 0.009 | 0.013 | 0.007 | 0.017 | 0.053 | 0.006 | 0.959 | 0.095 | 0.057 |
| Karamoja | 0.000 | -0.002 | 0.001 | 0.013 | 0.007 | 0.001 | 0.114 | 0.015 | 0.621 | 0.086 | 0.156 |
| West Nile | 0.005 | 0.011 | 0.017 | 0.018 | 0.009 | 0.009 | -0.003 | -0.010 | 0.663 | 0.023 | 0.085 |
| Western | 0.020 | 0.019 | 0.034 | 0.078 | 0.016 | 0.016 | 0.070 | 0.030 | 0.259 | 0.170 | 0.202 |
| Southwest | 0.016 | 0.007 | 0.015 | 0.039 | 0.014 | 0.020 | 0.034 | 0.022 | 0.117 | 0.044 | 0.024 |

**Table S11: Subnational vaccine equity metric for 2006: Wagstaff multivariate concentration index.**

| **Region** | **Vaccines and health outcomes** | | | | | | | | | | |
| --- | --- | --- | --- | --- | --- | --- | --- | --- | --- | --- | --- |
|  | **BCG** | **DPT1** | **DPT2** | **DPT3** | **OPV1** | **OPV2** | **OPV3** | **MCV1** | **ZERO** | **FULL** | **COMP** |
| Central 1 | 0.035 | 0.079 | 0.133 | 0.215 | 0.006 | 0.009 | 0.048 | 0.060 | 0.189 | 0.351 | 0.559 |
| Central 2 | 0.007 | 0.029 | 0.028 | 0.033 | 0.005 | -0.011 | 0.009 | 0.021 | 0.189 | 0.187 | 0.161 |
| Kampala | 0.007 | -0.009 | 0.006 | 0.038 | 0.008 | 0.032 | 0.052 | 0.005 | 0.024 | -0.016 | 0.197 |
| East central | 0.029 | 0.001 | 0.010 | 0.098 | 0.030 | 0.041 | 0.069 | 0.061 | -0.074 | 0.114 | 0.072 |
| Eastern | 0.016 | 0.035 | 0.073 | 0.075 | 0.009 | 0.000 | -0.024 | 0.008 | 0.386 | 0.023 | 0.025 |
| North | 0.007 | 0.048 | 0.057 | 0.061 | -0.001 | 0.011 | 0.028 | 0.011 | 0.449 | 0.131 | 0.128 |
| West Nile | 0.001 | 0.066 | 0.075 | 0.121 | 0.003 | 0.024 | 0.060 | 0.031 | 0.710 | -0.031 | -0.140 |
| Western | 0.017 | 0.025 | 0.054 | 0.056 | 0.011 | 0.029 | 0.045 | 0.027 | -0.027 | -0.003 | 0.032 |
| Southwest | 0.021 | 0.014 | 0.033 | 0.045 | 0.020 | 0.044 | 0.053 | 0.062 | 0.054 | 0.254 | 0.213 |

**Table S12: Subnational vaccine equity metric for 2000: Wagstaff multivariate concentration index.**

| **Region (2000)** | **Vaccines and health outcomes** | | | | | | | | | | |
| --- | --- | --- | --- | --- | --- | --- | --- | --- | --- | --- | --- |
|  | **BCG** | **DPT1** | **DPT2** | **DPT3** | **OPV1** | **OPV2** | **OPV3** | **MCV1** | **ZERO** | **FULL** | **COMP** |
| Central | 0.099 | 0.090 | 0.112 | 0.154 | 0.079 | 0.092 | 0.122 | 0.096 | 0.443 | 0.136 | 0.101 |
| Eastern | 0.053 | 0.046 | 0.069 | 0.120 | 0.009 | 0.033 | 0.054 | 0.043 | 0.336 | 0.121 | 0.114 |
| Northern | 0.024 | 0.012 | 0.050 | 0.072 | 0.004 | 0.006 | 0.018 | 0.025 | 0.029 | 0.074 | 0.055 |
| Western | 0.053 | 0.049 | 0.053 | 0.083 | 0.037 | 0.049 | 0.078 | 0.070 | 0.344 | 0.085 | 0.057 |

**Table S13: Subnational vaccine equity metric for 2016: Erreyger multivariate concentration index.**

| **Region** | **Vaccines and health outcomes** | | | | | | | | | | | | | |
| --- | --- | --- | --- | --- | --- | --- | --- | --- | --- | --- | --- | --- | --- | --- |
|  | **BCG** | **DPT1** | **DPT2** | **DPT3** | **OPV1** | **OPV2** | **OPV3** | **PCV1** | **PCV2** | **PCV3** | **MCV1** | **ZERO** | **FULL** | **COMP** |
| Kampala | 0.031 | 0.044 | 0.118 | 0.191 | 0.029 | 0.131 | 0.134 | 0.064 | 0.150 | 0.151 | 0.095 | 0.007 | 0.093 | 0.226 |
| South Buganda | 0.107 | 0.094 | 0.128 | 0.157 | 0.085 | 0.093 | 0.142 | 0.159 | 0.160 | 0.173 | 0.176 | 0.033 | 0.197 | 0.188 |
| North Buganda | 0.103 | 0.098 | 0.124 | 0.149 | 0.095 | 0.141 | 0.023 | 0.150 | 0.224 | 0.232 | 0.143 | 0.019 | 0.113 | 0.144 |
| Busoga | 0.034 | 0.061 | 0.053 | 0.118 | 0.019 | 0.067 | 0.068 | 0.016 | 0.065 | 0.151 | 0.067 | -0.014 | 0.076 | -0.002 |
| Bukedi | 0.029 | 0.006 | 0.097 | 0.090 | -0.001 | 0.086 | 0.106 | -0.018 | 0.078 | 0.089 | 0.109 | 0.012 | 0.108 | 0.101 |
| Bugisu | 0.018 | 0.062 | 0.081 | 0.055 | 0.045 | 0.096 | 0.089 | 0.110 | 0.075 | 0.071 | 0.013 | NA | 0.158 | 0.222 |
| Teso | -0.003 | -0.046 | -0.018 | -0.032 | -0.015 | 0.014 | 0.010 | 0.022 | 0.038 | 0.045 | 0.052 | -0.001 | 0.080 | 0.084 |
| Karamoja | 0.020 | 0.016 | -0.025 | -0.051 | 0.015 | 0.011 | -0.064 | 0.067 | 0.094 | 0.023 | -0.022 | 0.000 | 0.081 | 0.067 |
| Lango | 0.021 | 0.026 | 0.063 | 0.079 | 0.018 | 0.068 | 0.016 | 0.018 | 0.058 | 0.029 | 0.077 | 0.008 | 0.033 | -0.136 |
| Acholi | 0.001 | 0.069 | 0.076 | 0.050 | 0.070 | 0.069 | 0.063 | 0.135 | 0.048 | 0.109 | 0.162 | 0.015 | 0.131 | 0.120 |
| West Nile | 0.049 | 0.028 | 0.040 | 0.070 | 0.028 | 0.057 | 0.124 | 0.003 | 0.028 | 0.061 | 0.041 | 0.023 | 0.155 | 0.196 |
| Bunyoro | 0.093 | 0.071 | 0.084 | 0.122 | 0.054 | 0.049 | 0.079 | 0.127 | 0.084 | 0.119 | 0.072 | 0.043 | 0.092 | 0.051 |
| Tooro | 0.023 | 0.054 | 0.099 | 0.064 | 0.051 | 0.081 | 0.013 | 0.049 | 0.059 | 0.055 | 0.083 | 0.000 | 0.067 | 0.064 |
| Ankole | 0.085 | 0.079 | 0.089 | 0.113 | 0.081 | 0.079 | 0.139 | 0.068 | 0.111 | 0.081 | 0.061 | 0.011 | 0.108 | 0.276 |
| Kigezi | 0.068 | 0.040 | 0.105 | 0.093 | 0.053 | 0.049 | 0.054 | 0.081 | 0.099 | 0.093 | 0.152 | NA | -0.014 | 0.183 |

**Table S14: Subnational vaccine equity metric for 2011: Erreyger multivariate concentration index.**

| **Region** | **Vaccines and health outcomes** | | | | | | | | | | |
| --- | --- | --- | --- | --- | --- | --- | --- | --- | --- | --- | --- |
|  | **BCG** | **DPT1** | **DPT2** | **DPT3** | **OPV1** | **OPV2** | **OPV3** | **MCV1** | **ZERO** | **FULL** | **COMP** |
| Kampala | 0.030 | 0.020 | 0.056 | 0.114 | 0.052 | 0.131 | 0.076 | 0.100 | -0.002 | 0.220 | 0.024 |
| Central 1 | 0.148 | 0.109 | 0.165 | 0.197 | 0.050 | 0.102 | 0.119 | 0.136 | 0.017 | 0.268 | 0.299 |
| Central 2 | 0.074 | 0.081 | 0.115 | 0.193 | 0.063 | 0.110 | 0.147 | 0.114 | 0.010 | 0.214 | 0.290 |
| East central | 0.038 | 0.014 | 0.080 | 0.153 | 0.004 | 0.053 | 0.071 | 0.103 | 0.007 | 0.216 | 0.180 |
| Eastern | 0.009 | 0.045 | 0.040 | 0.039 | 0.035 | 0.072 | 0.009 | 0.109 | NA | 0.019 | -0.045 |
| North | 0.052 | 0.021 | 0.032 | 0.040 | 0.026 | 0.056 | 0.119 | 0.017 | 0.011 | 0.209 | 0.130 |
| Karamoja | -0.001 | -0.006 | 0.002 | 0.040 | 0.025 | 0.004 | 0.188 | 0.046 | 0.011 | 0.123 | 0.208 |
| West Nile | 0.020 | 0.042 | 0.059 | 0.055 | 0.034 | 0.029 | -0.008 | -0.029 | 0.011 | 0.050 | 0.216 |
| Western | 0.074 | 0.070 | 0.110 | 0.204 | 0.057 | 0.053 | 0.175 | 0.088 | 0.008 | 0.330 | 0.439 |
| Southwest | 0.053 | 0.022 | 0.046 | 0.108 | 0.047 | 0.062 | 0.090 | 0.053 | 0.011 | 0.110 | 0.074 |

**Table S15: Subnational vaccine equity metric for 2006: Erreyger multivariate concentration index.**

| **Region** | **Vaccines and health outcomes** | | | | | | | | | | |
| --- | --- | --- | --- | --- | --- | --- | --- | --- | --- | --- | --- |
|  | **BCG** | **DPT1** | **DPT2** | **DPT3** | **OPV1** | **OPV2** | **OPV3** | **MCV1** | **ZERO** | **FULL** | **COMP** |
| Central 1 | 0.105 | 0.102 | 0.119 | 0.105 | 0.017 | 0.021 | 0.084 | 0.129 | 0.025 | 0.135 | 0.270 |
| Central 2 | 0.022 | 0.039 | 0.033 | 0.027 | 0.017 | -0.032 | 0.019 | 0.052 | 0.021 | 0.092 | 0.109 |
| Kampala | 0.025 | -0.015 | 0.010 | 0.045 | 0.028 | 0.097 | 0.105 | 0.013 | 0.001 | -0.008 | 0.100 |
| East central | 0.103 | 0.002 | 0.013 | 0.090 | 0.102 | 0.116 | 0.129 | 0.139 | -0.005 | 0.039 | 0.036 |
| Eastern | 0.057 | 0.058 | 0.103 | 0.071 | 0.031 | 0.000 | -0.051 | 0.020 | 0.015 | 0.014 | 0.021 |
| North | 0.024 | 0.095 | 0.097 | 0.078 | -0.002 | 0.034 | 0.056 | 0.032 | 0.007 | 0.073 | 0.102 |
| West Nile | 0.005 | 0.102 | 0.103 | 0.127 | 0.011 | 0.076 | 0.125 | 0.081 | 0.009 | -0.017 | -0.084 |
| Western | 0.061 | 0.045 | 0.083 | 0.053 | 0.039 | 0.090 | 0.104 | 0.074 | -0.001 | -0.001 | 0.018 |
| Southwest | 0.069 | 0.019 | 0.038 | 0.037 | 0.065 | 0.134 | 0.123 | 0.157 | 0.007 | 0.117 | 0.149 |

**Table S16: Subnational vaccine equity metric for 2000: Erreyger multivariate concentration index.**

| **Region (2000)** | **Vaccines and health outcomes** | | | | | | | | | | |
| --- | --- | --- | --- | --- | --- | --- | --- | --- | --- | --- | --- |
|  | **BCG** | **DPT1** | **DPT2** | **DPT3** | **OPV1** | **OPV2** | **OPV3** | **MCV1** | **ZERO** | **FULL** | **COMP** |
| Central | 0.286 | 0.247 | 0.252 | 0.262 | 0.234 | 0.230 | 0.206 | 0.203 | 0.179 | 0.172 | 0.170 |
| Eastern | 0.177 | 0.143 | 0.177 | 0.220 | 0.029 | 0.093 | 0.119 | 0.092 | 0.039 | 0.187 | 0.209 |
| Northern | 0.075 | 0.035 | 0.123 | 0.124 | 0.014 | 0.016 | 0.036 | 0.057 | 0.008 | 0.100 | 0.089 |
| Western | 0.173 | 0.162 | 0.159 | 0.210 | 0.130 | 0.156 | 0.207 | 0.187 | 0.063 | 0.181 | 0.155 |

**Table S17: National coverage estimates by sociodemographic group in 2016.**

| **Characteristic** | **Vaccines and health outcomes** | | | | | | | | | | | | | |
| --- | --- | --- | --- | --- | --- | --- | --- | --- | --- | --- | --- | --- | --- | --- |
|  | **BCG** | **DPT1** | **DPT2** | **DPT3** | **OPV1** | **OPV2** | **OPV3** | **MCV1** | **PCV1** | **PCV2** | **PCV3** | **ZERO** | **FULL** | **COMP** |
| **Urban/rural** |  |  |  |  |  |  |  |  |  |  |  |  |  |  |
| Urban | 96.2% | 90.4% | 82.1% | 71.0% | 87.1% | 76.9% | 58.2% | 65.2% | 81.3% | 73.4% | 61.4% | 1.2% | 41.6% | 40.6% |
| Rural | 93.7% | 87.2% | 79.4% | 67.9% | 84.8% | 75.8% | 59.1% | 58.0% | 76.1% | 67.5% | 56.1% | 1.5% | 40.5% | 38.6% |
|  |  |  |  |  |  |  |  |  |  |  |  |  |  |  |
| **Wealth quintiles** |  |  |  |  |  |  |  |  |  |  |  |  |  |  |
| 1 – Poorest | 94.0% | 88.1% | 81.0% | 69.3% | 85.1% | 77.7% | 61.7% | 56.7% | 76.3% | 68.8% | 56.5% | 1.3% | 40.8% | 37.9% |
| 2 – Lower middle | 94.3% | 87.9% | 78.0% | 66.8% | 84.0% | 73.8% | 57.7% | 57.9% | 77.5% | 68.0% | 56.3% | 1.2% | 40.3% | 38.0% |
| 3 – Middle | 94.3% | 86.6% | 78.6% | 65.8% | 84.7% | 76.0% | 58.6% | 59.1% | 75.1% | 67.1% | 54.1% | 1.4% | 41.6% | 41.9% |
| 4 – Upper middle | 93.0% | 88.0% | 81.5% | 70.7% | 85.9% | 77.3% | 60.8% | 59.5% | 76.2% | 67.9% | 58.6% | 1.7% | 40.2% | 40.3% |
| 5 – Wealthiest | 95.4% | 88.9% | 80.9% | 70.5% | 84.7% | 75.3% | 55.6% | 65.1% | 80.7% | 72.0% | 60.8% | 1.5% | 40.9% | 37.6% |
|  |  |  |  |  |  |  |  |  |  |  |  |  |  |  |
| **Maternal education** |  |  |  |  |  |  |  |  |  |  |  |  |  |  |
| No education | 92.0% | 84.9% | 77.3% | 64.9% | 83.9% | 74.0% | 57.5% | 57.0% | 75.1% | 66.7% | 54.2% | 2.0% | 39.6% | 36.9% |
| Primary education | 93.8% | 87.3% | 78.8% | 66.9% | 84.4% | 74.9% | 58.3% | 57.2% | 75.4% | 66.5% | 54.8% | 1.5% | 39.0% | 37.5% |
| Secondary education | 95.0% | 89.4% | 82.4% | 71.9% | 87.1% | 77.8% | 61.2% | 64.8% | 80.2% | 72.2% | 62.0% | 1.2% | 43.3% | 41.6% |
| Tertiary education | 98.6% | 92.7% | 86.2% | 78.6% | 89.2% | 82.9% | 62.5% | 67.4% | 86.2% | 80.2% | 68.0% | 0.6% | 49.8% | 50.8% |
|  |  |  |  |  |  |  |  |  |  |  |  |  |  |  |
| **Sex of the child** |  |  |  |  |  |  |  |  |  |  |  |  |  |  |
| Male | 94.1% | 87.7% | 80.0% | 68.6% | 85.7% | 75.8% | 59.0% | 59.6% | 76.9% | 68.3% | 57.2% | 1.4% | 41.4% | 38.5% |
| Female | 94.3% | 88.1% | 80.0% | 68.6% | 84.8% | 76.3% | 58.9% | 59.5% | 77.5% | 69.2% | 57.3% | 1.4% | 40.1% | 39.6% |
|  |  |  |  |  |  |  |  |  |  |  |  |  |  |  |
| **Health insurance** |  |  |  |  |  |  |  |  |  |  |  |  |  |  |
| Yes | 94.2% | 87.8% | 86.6% | 68.5% | 85.3% | 75.9% | 58.8% | 59.4% | 77.1% | 68.6% | 57.1% | 1.4% | 40.7% | 38.9% |
| No | 98.9% | 93.2% | 79.9% | 74.9% | 86.6% | 81.8% | 69.3% | 68.9% | 82.6% | 76.4% | 62.8% | 1.1% | 48.3% | 46.7% |

**Table S18: National coverage estimates by sociodemographic group in 2000.**

| **Characteristic^1^** | **Vaccines and health outcomes** | | | | | | | | | | |
| --- | --- | --- | --- | --- | --- | --- | --- | --- | --- | --- | --- |
|  | **BCG** | **DPT1** | **DPT2** | **DPT3** | **OPV1** | **OPV2** | **OPV3** | **MCV1** | **ZERO** | **FULL** | **COMP** |
| **Urban/rural** |  |  |  |  |  |  |  |  |  |  |  |
| Urban | 87.1% | 81.3% | 70.3% | 56.5% | 84.5% | 74.0% | 55.3% | 62.5% | 3.1% | 44.1% | 54.4% |
| Rural | 76.2% | 74.0% | 61.7% | 45.5% | 80.4% | 69.8% | 52.2% | 55.1% | 7.0% | 37.4% | 47.1% |
|  |  |  |  |  |  |  |  |  |  |  |  |
| **Wealth quintiles** |  |  |  |  |  |  |  |  |  |  |  |
| 1 – Poorest | 78.1% | 75.1% | 60.5% | 44.3% | 82.7% | 71.4% | 54.4% | 55.1% | 5.7% | 36.6% | 43.9% |
| 2 – Lower middle | 81.2% | 80.9% | 70.9% | 53.4% | 74.3% | 77.6% | 58.5% | 66.6% | 2.8% | 43.7% | 55.6% |
| 3 – Middle | 80.2% | 77.5% | 65.9% | 50.2% | 81.8% | 71.7% | 58.0% | 56.7% | 4.9% | 41.9% | 48.8% |
| 4 – Upper middle | 68.6% | 67.6% | 57.4% | 43.8% | 73.1% | 63.0% | 46.0% | 51.8% | 12.6% | 35.7% | 46.4% |
| 5 – Wealthiest | 79.3% | 75.1% | 62.4% | 45.4% | 81.3% | 70.3% | 49.9% | 54.2% | 6.0% | 36.2% | 47.9% |
|  |  |  |  |  |  |  |  |  |  |  |  |
| **Maternal education** |  |  |  |  |  |  |  |  |  |  |  |
| No education | 71.4% | 69.8% | 57.4% | 41.4% | 77.6% | 66.2% | 49.3% | 53.0% | 9.2% | 33.9% | 43.8% |
| Primary education | 77.5% | 75.1% | 62.8% | 46.3% | 81.3% | 70.7% | 52.9% | 55.1% | 6.3% | 38.3% | 48.4% |
| Secondary education | 87.4% | 82.1% | 70.9% | 56.1% | 83.7% | 74.0% | 54.8% | 63.8% | 3.4% | 42.9% | 51.4% |
| Tertiary education | 97.1% | 92.8% | 86.6% | 79.2% | 92.0% | 88.6% | 71.1% | 81.5% | 0.5% | 59.9% | 66.7% |
|  |  |  |  |  |  |  |  |  |  |  |  |
| **Sex of the child** |  |  |  |  |  |  |  |  |  |  |  |
| Male | 77.9% | 74.8% | 63.1% | 46.2% | 81.5% | 70.3% | 52.2% | 56.1% | 6.2% | 38.3% | 48.4% |
| Female | 76.9% | 74.8% | 62.4% | 47.2% | 80.2% | 70.2% | 52.9% | 55.8% | 7.0% | 37.8% | 47.4% |

Notes: ^1.^ Everyone was uninsured (as defined in the DHS questionnaire).

**Table S19: Coverage estimates by composite quintile and absolute equity gap in 2016.**

| **Vaccine or health outcome ^1^** | **Coverage** | **Composite quintiles** | | **AEG** |
| --- | --- | --- | --- | --- |
|  |  | **1: Most disadvantaged** | **5: Most privileged** |  |
| BCG | 94.2% | 88.3% | 98.2% | 0.098 |
| DPT1 | 92.7% | 81.1% | 91.8% | 0.106 |
| DPT2 | 86.8% | 72.2% | 86.8% | 0.146 |
| DPT3 | 76.8% | 59.1% | 77.6% | 0.183 |
| POLIO1 | 89.7% | 79.0% | 89.6% | 0.107 |
| POLIO2 | 82.5% | 68.0% | 83.3% | 0.153 |
| POLIO3 | 65.9% | 50.8% | 69.6% | 0.185 |
| PCV1 | 81.4% | 67.1% | 68.9% | 0.177 |
| PCV2 | 74.6% | 57.7% | 84.8% | 0.215 |
| PCV3 | 64.1% | 46.5% | 79.6% | 0.197 |
| MCV1 | 82.7% | 50.7% | 66.5% | 0.180 |
| ZERO | 2.2% | 2.8% | 0.2% | 0.027 |
| FULL | 40.8% | 30.3% | 53.6% | 0.230 |
| COMPLETE | 39.0% | 26.3% | 53.4% | 0.267 |

**Table S20: Coverage estimates by composite quintile and absolute equity gap in 2011.**

| **Vaccine or health outcome ^1^** | **Coverage** | **Composite quintiles** | | **AEG** |
| --- | --- | --- | --- | --- |
|  |  | **1: Most disadvantaged** | **5: Most privileged** |  |
| BCG | 92.1% | 83.9% | 98.3% | 0.141 |
| DPT1 | 89.0% | 79.5% | 94.3% | 0.147 |
| DPT2 | 80.8% | 69.4% | 88.2% | 0.181 |
| DPT3 | 66.9% | 50.3% | 76.5% | 0.263 |
| POLIO1 | 89.0% | 81.8% | 93.4% | 0.120 |
| POLIO2 | 79.5% | 69.8% | 86.5% | 0.165 |
| POLIO3 | 57.3% | 44.0% | 66.2% | 0.221 |
| MCV1 | 70.9% | 60.2% | 76.3% | 0.157 |
| ZERO | 0.8% | 1.9% | 0.0% | 0.019 |
| FULL | 48.9% | 31.3% | 63.3% | 0.321 |
| COMPLETE | 54.8% | 33.4% | 71.4% | 0.379 |

**Table S21: Coverage estimates by composite quintile and absolute equity gap in 2006.**

| **Vaccine or health outcome ^1^** | **Coverage** | **Composite quintiles** | | **AEG** |
| --- | --- | --- | --- | --- |
|  |  | **1: Most disadvantaged** | **5: Most privileged** |  |
| BCG | 88.7% | 79.0% | 95.2% | 0.162 |
| DPT1 | 41.2% | 30.7% | 49.9% | 0.185 |
| DPT2 | 35.5% | 24.1% | 44.1% | 0.196 |
| DPT3 | 24.5% | 15.5% | 31.5% | 0.159 |
| POLIO1 | 87.5% | 79.4% | 91.7% | 0.123 |
| POLIO2 | 76.9% | 65.2% | 82.2% | 0.172 |
| POLIO3 | 53.8% | 43.5% | 61.1% | 0.169 |
| MCV1 | 68.4% | 54.9% | 72.7% | 0.177 |
| ZERO | 1.6% | 3.3% | 0.0% | 0.032 |
| FULL | 12.5% | 7.4% | 19.1% | 0.117 |
| COMPLETE | 15.8% | 10.8% | 27.4% | 0.167 |

**Table S22: Coverage estimates by composite quintile and absolute equity gap in 2000.**

| **Vaccine or health outcome ^1^** | **Coverage** | **Composite quintiles** | | **AEG** |
| --- | --- | --- | --- | --- |
|  |  | **1: Most disadvantaged** | **5: Most privileged** |  |
| BCG | 77.4% | 62.6% | 91.1% | 0.269 |
| DPT1 | 76.8% | 63.0% | 86.8% | 0.234 |
| DPT2 | 65.6% | 48.2% | 80.1% | 0.305 |
| DPT3 | 49.7% | 34.8% | 70.6% | 0.358 |
| POLIO1 | 83.0% | 68.4% | 88.9% | 0.185 |
| POLIO2 | 73.5% | 57.6% | 83.6% | 0.261 |
| POLIO3 | 56.0% | 38.5% | 70.9% | 0.317 |
| MCV1 | 67.8% | 49.5% | 73.0% | 0.234 |
| ZERO | 8.4% | 13.5% | 1.1% | 0.123 |
| FULL | 38.1% | 28.1% | 28.1% | 0.291 |
| COMPLETE | 47.9% | 34.9% | 34.9% | 0.363 |

**Reference**

1. Erreygers G. Correcting the concentration index. J Health Econ. 2009;28(2):504-15.

2. Wagstaff A. The concentration index of a binary outcome revisited. Health Econ. 2011;20(10):1155-60.
